# Unraveling nonlinear electrophysiologic processes in the human visual system with full dimension spectral analysis

**DOI:** 10.1038/s41598-019-53286-z

**Published:** 2019-11-15

**Authors:** Kien Trong Nguyen, Wei-Kuang Liang, Victor Lee, Wen-Sheng Chang, Neil G. Muggleton, Jia-Rong Yeh, Norden E. Huang, Chi-Hung Juan

**Affiliations:** 1 Institute of Cognitive Neuroscience, National Central University, Taoyuan, Taiwan; 2 Brain Research Center, National Central University, Taoyuan, Taiwan; 3 Institute of Cognitive Neuroscience, University College London, London, UK; 4 Department of Psychology, Goldsmiths, University of London, London, UK; 5 Data Analysis and Application Laboratory, The First Institute of Oceanography, Qingdao, China; 6 Pilot National Laboratory of Marine Science and Technology, Qingdao, China

**Keywords:** Electroencephalography - EEG, Cognitive neuroscience, Visual system

## Abstract

Natural sensory signals have nonlinear structures dynamically composed of the carrier frequencies and the variation of the amplitude (i.e., envelope). How the human brain processes the envelope information is still poorly understood, largely due to the conventional analysis failing to quantify it directly. Here, we used a recently developed method, Holo-Hilbert spectral analysis, and steady-state visually evoked potential collected using electroencephalography (EEG) recordings to investigate how the human visual system processes the envelope of amplitude-modulated signals, in this case with a 14 Hz carrier and a 2 Hz envelope. The EEG results demonstrated that in addition to the fundamental stimulus frequencies, 4 Hz amplitude modulation residing in 14 Hz carrier and a broad range of carrier frequencies covering from 8 to 32 Hz modulated by 2 Hz amplitude modulation are also found in the two-dimensional frequency spectrum, which have not yet been recognized before. The envelope of the stimulus is also found to dominantly modulate the response to the incoming signal. The findings thus reveal that the electrophysiological response to amplitude-modulated stimuli is more complex than could be revealed by, for example, Fourier analysis. This highlights the dynamics of neural processes in the visual system.

## Introduction

The brain processes natural sensory stimuli in a dynamic, non-stationary and nonlinear way^1–3^. Such naturalistic stimuli are often a combination of several sinusoidal oscillations with various frequencies generating a signal with an embedded envelope that modulates the amplitude of the carrier frequency. Such slow time-varying envelopes can be found in most natural auditory, visual and somatosensory stimuli. For example, the electrosensory system of each weakly electric fish generates a stable carrier wave electric organ discharge. However, when two electric fish meet each other, their electric organ discharges are superimposed and then generate an amplitude-modulated (AM) signal in the electric field^4^. The envelopes of the AM signals are extracted and processed by a specialized neural system allowing the weakly electric fish to obtain crucial information which is critical for interaction with conspecifics and for survival^4,5^. Studies of mammals have also shown cortical cells process envelope and carrier features in, for example, the auditory cortex of monkeys and the visual cortex of cats^6–8^. Nevertheless, how to investigate the neural processing of nonlinear stimuli by using a noninvasive technique (e.g. electroencephalography; EEG) is one of the major challenges in the study of human brain function. The human visual system is thought as a well-suited system for investigating nonlinear processing in the brain because a periodic sinusoidal flicker can be used to reliably elicit a steady-state visually evoked potential (SSVEP) with stable amplitude and phase over time^9^. In the frequency domain, using Fourier transform, this SSVEP response presents not only narrowband peaks associated with the stimulus frequencies but also at their so called “harmonic components” (i.e., responses at frequencies that are multiples of the stimulus frequencies). Thus, these harmonic components represent nonlinear responses of the visual system^10^. However, the sinusoidal flicker is a stimulus that does not contain any complex attributes seen in nature, which limits the understanding of nonlinear information processing^11^. To obtain a deep understanding of nonlinear processes, studies have attempted to employ AM signals which could be treated as a combination of two or more sinusoidal oscillations^11–13^, or a simple nonlinear multiplicative modulation^14^. These AM signals closely mimic natural visual stimuli. In the visual system, when more than one frequency input is applied, evoked responses can be derived by sums and differences of the frequency inputs, which is called intermodulation (IM) as revealed by Fast Fourier transform (FFT)^11–13,15–18^. Despite the considerable number of studies investigating nonlinear responses by observing these IM components, how AM stimuli are processed remains largely unknown in visual systems. One reason is that the envelope of AM stimuli is a non-Fourier component^19,20^, so it is difficult to capture the neural responses of the envelope using conventional time-series data decomposition methods (e.g., Fourier transform). Specifically, the Fourier transform is based on additive expansion which ignores the neural signal waveform shapes and the amplitude modulation of neural oscillations^14,21–23^. Therefore, nonlinear neural processing of time-varying envelopes are not examined in most studies. To delve deeper into understanding how the visual system processes the temporal envelope of AM signals and how envelope responses interact with other neural processes, nonlinear analytical methods are required to quantify the evoked response of envelope and its nonlinear characteristics. Huang and colleagues have recently introduced Holo-Hilbert Spectral Analysis (HHSA), an advanced Hilbert Huang Transform-derived analytical tool capable of processing nonlinear and nonstationary signals^14^. HHSA, which is a nonlinear analysis tool based on the Empirical Mode Decomposition (EMD), provides a fully informational and high-dimensional frequency representation of data resulting from non-stationary and nonlinear processes. That is, both the carrier frequencies (*f*_*c*_) and the amplitude modulation frequencies (*f*_*am*_) in the signal can be examined simultaneously in the Holo-Hilbert spectrum (HHS)^14^ (see the illustration of HHSA in Supplementary Fig. S1). Although the nonlinear modulation view has been comprehensively investigated in the level of frequency modulation^24–26^, the nonlinearity of amplitude modulation remains elusive and the current method provides a direction for this line of research. Furthermore, this comprehensive approach allows the investigation of the carrier frequencies and the amplitude modulation frequencies and also their interaction in neural oscillations.

Therefore, the main goal of this work was to elucidate the visual nonlinear neural processing of the envelope in AM signals (experiment 1) and to explore how the envelope interacts with other incoming extrinsic signals simultaneously (experiment 2) in two-dimensional frequency representations by using HHSA. In comparison to previous visual studies, it is essential to understand the fundamental and nonlinear visual processes from the two eyes, in which performance for both eyes is enhanced compared to one eye in both psychophysical and electrophysiological studies^27,28^. Thus, experiment 1 was designed to explore these visual processes for both binocular (presenting identical visual stimuli to both eyes) and monocular viewing conditions (presenting the stimuli to one eye). In addition, previous studies^29,30^ have found that dichoptic stimulation differences generate competition between opposite monocular pathways. Therefore, experiment 2 aimed to further understand the role of the envelope in interocular interactions, with how AM signals presented to one eye affects a uniform sine-wave signal presented to the other eye.

## Results

### Experiment 1: Monocular and binocular stimulation

Holo-Hilbert spectral analysis is mainly based on the EMD to resolve the identification of intrinsic amplitude modulations by representing the data in multiple dimensions (i.e., frequency of amplitude modulation, frequency of carrier and time). Therefore, the masking EMD, which was applied in HHSA in this study, should successfully decompose the multi-component signal into single modes, as known as intrinsic mode functions (IMFs), which naturally retain the physical meaning of the signal without suffering mode-mixing. Thus, before applying this decomposition method in the real data (photodiode and electrophysiological data), it was firstly validated in the simulation using two different amplitude-modulated signals with addition of robust noise (signal-to-noise ratio (db) was controlled to be zero), for observing the physical meaning in the decomposed signal. The simulation data consisted of the sum of two amplitude-modulated signals, which are 2 Hz modulated 7 Hz and 14 Hz, and robust noise. The results of masking EMD showed that the signal could be successfully decomposed into each IMF retaining the physical meaning in the condition of strong noise (see illustration in SI) and could be comparable to other decomposition methods (e.g., Nonlinear Mode Decomposition^31^ and Swarm Decomposition^32^, which are new methods and proven to be noise robust) (see the illustration in Supplementary Figs S2–S3). Moreover, the decomposed components (i.e., IMFs) are successfully separated in different frequency bands without the mode-mixing. Therefore, the merit of EMD had been validated and can be successfully applied in HHSA to obtain true phenomena in the real data.

**Figure 1:**
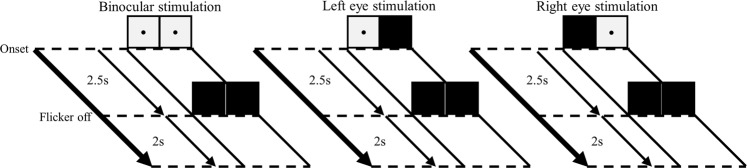
Various light flicker stimuli were presented to participants in a randomized manner with a time duration of 2.5 s. After stimulation, participants could close their eyes and rest until the next trial. Light flicker stimulation could be presented to both eyes (binocular), only to the left or only to the right eye (monocular).

**Figure 2:**
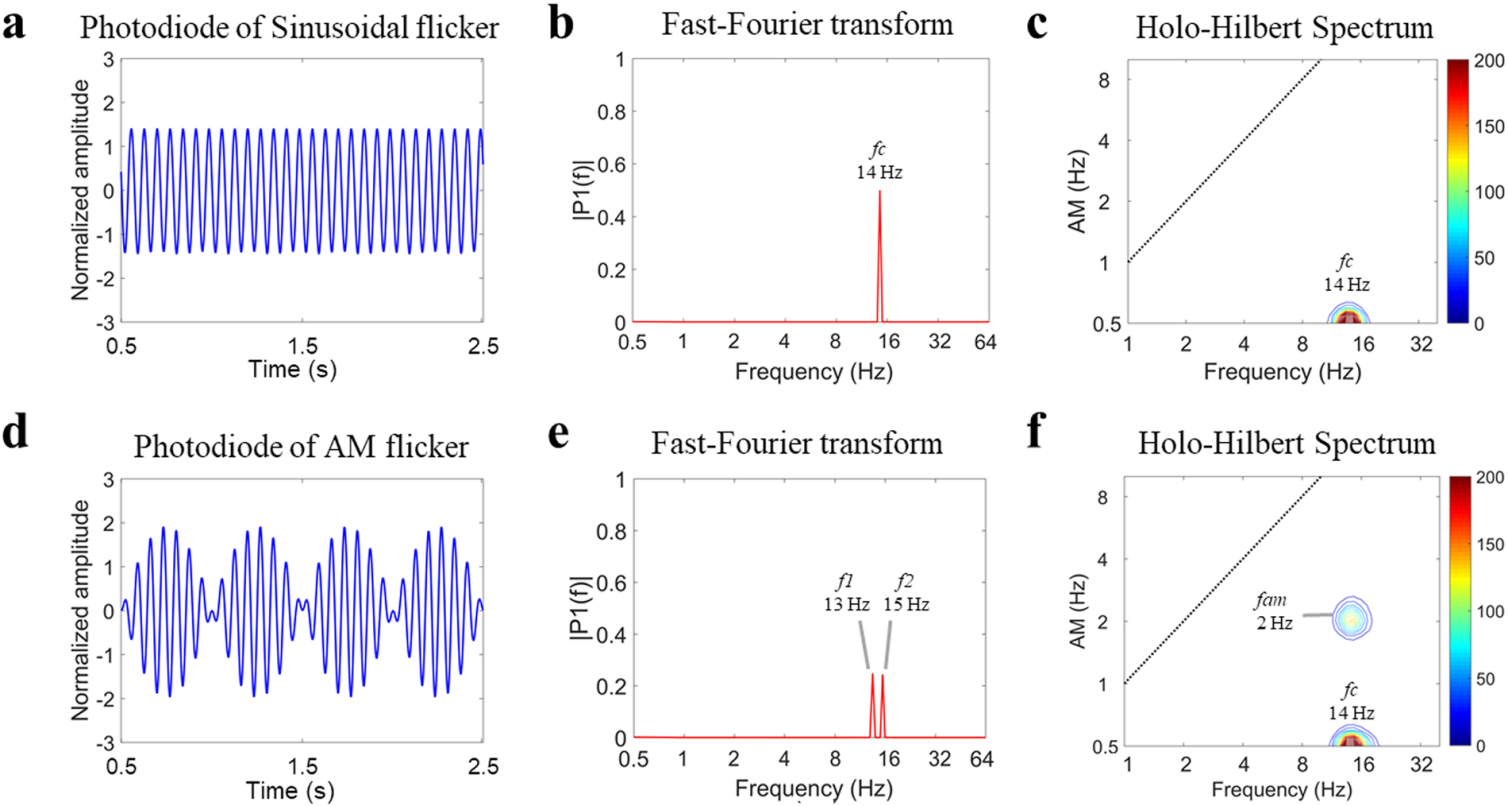
(Experiment 1) Photodiode recordings of uniform sinusoid and AM signals revealed in the frequency domain (using FFT) and two-dimensional frequency spectrum (using HHSA, see Supplementary Material and methods). (**a**) The photodiode response to 14 Hz sinusoidal signal. (**b**) The FFT was applied to 2-second photodiode recordings of sinusoidal flicker showing the strong amplitude spectrum at 14 Hz. (**c**) The HHS showed the fundamental 14 Hz carrier frequency and shows no envelope modulations. (**d**) The waveform of 2 Hz envelope modulating 14 Hz AM stimulus captured by the photodiode. (**e**) The FFT analysis revealed 13 and 15 Hz peaks. However, the Fourier spectrum did not show the amplitude modulation frequency. (**f**) The HHSA provided separately the amplitude spectrum of a 2 Hz *f*_*am*_ modulating 14 Hz *f*_*c*_ and 14 Hz carrier in two-dimensional frequency spectra. In subfigures (**c**,**f**) the x-axis denotes the fast-changing carrier intra-mode frequency variations (*f*_*c*_), and the y-axis denotes the slow-changing envelope inter-mode frequency variations (*f*_*am*_). At 0.5 Hz y-axis, this x-axis is the summed amplitude of carrier frequencies over time. The frequency axes represent dyadic frequency, in which the edges of dyadic frequency bins are defined by the formula 2^n^ (where n = −1, 0, 1, 2, 3, 4, 5).

**Figure 3:**
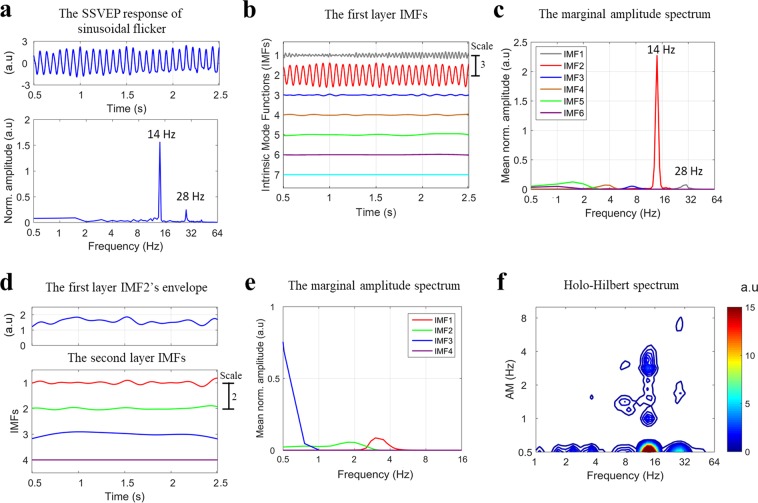
(Experiment 1) Illustration of two layer IMFs from a SSVEP response elicited by sinusoidal flicker in binocular stimulation for single subject at the Oz channel. (**a**) The SSVEP response in the time domain (top panel) and in the frequency domain (analyzed with FFT) (bottom panel). In FFT, SSVEP amplitude showed the peaks at the fundamental frequency (i.e., 14 Hz) and a nonlinear component at frequency-doubling (i.e., 28 Hz). (**b**) The first layer IMFs of SSVEP response decomposed by masking EMD contains 7 IMFs during this sinusoidal flicker condition. (**c**) The marginal amplitude spectrum of Hilbert-Huang transform of first layer IMFs. The strong amplitude of the second IMF (IMF2; peaked at around 14 Hz) in this spectrum was observed at stimulus frequency (14 Hz). (**d**) The masking EMD was applied to the envelope of IMF2 of the first layer IMFs (top panel) to obtain the second layer IMFs (bottom panel), which contains 4 IMFs. (**e**) The marginal amplitude spectrum of HHT of the second layer IMFs. (**f**) The Holo-Hilbert spectrum of SSVEP response induced by sinusoidal flicker. In two-dimensional frequency spectrum, the HHS showed the strong amplitude at 14 Hz fundamental stimulus frequency and its frequency-doubling (i.e., 28 Hz). Some weak amplitude of 1 to 4 Hz modulating 14 Hz were observed. The x-axis represents the carrier frequency (*f*_*c*_), and the y-axis represents the amplitude modulation frequency (*f*_*am*_). At 0.5 Hz y-axis, this x-axis is the summed amplitude of carrier frequencies over time. The frequency axes represent in a dyadic frequency scale.

**Figure 4:**
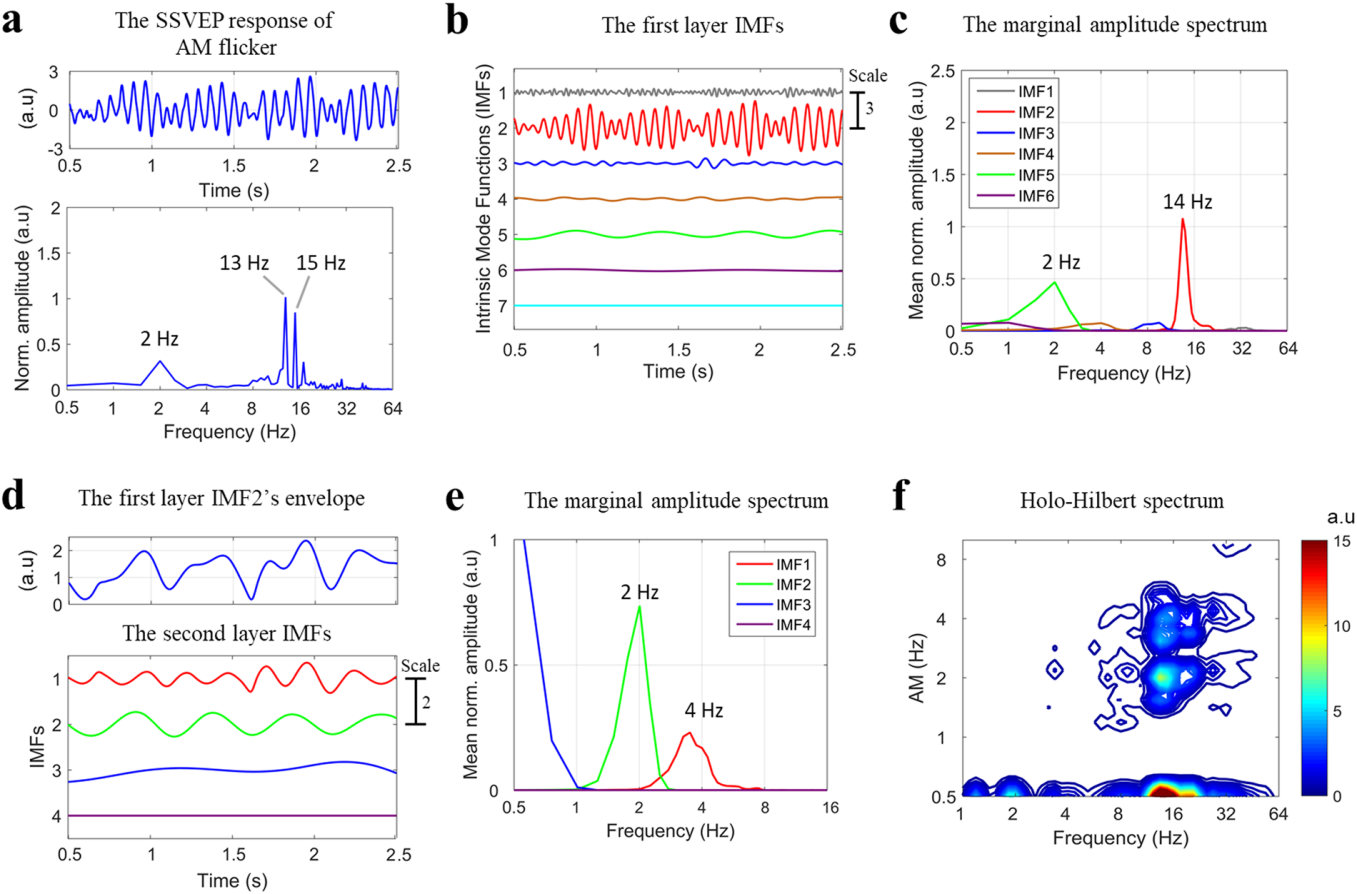
(Experiment 1) Illustration of two layer IMFs from an SSVEP elicited by AM flicker in binocular stimulation for a single subject at the Oz channel. (**a**) The SSVEP response in the time domain (top panel) and frequency domain (FFT) (bottom panel). In FFT, the SSVEP spectrum showed a series of nonlinear intermodulation components, in which frequencies could be expressed by the equation: m*f_1_ ± n*f_2_, where *f*_1_ (13 Hz) and *f*_2_ (15 Hz) were the fundamental frequencies, and m and n represented positive integers. (**b**) The first layer IMFs of SSVEP response decomposed by masking EMD contains 7 IMFs during the AM flicker condition. (**c**) The marginal amplitude spectrum of Hilbert-Huang transform of the first layer IMFs. The strong amplitude of the second IMF (IMF2; peaked at around 14 Hz) in this spectrum was observed at stimulus frequency (14 Hz). The amplitude spectrum of the fifth IMF was also dominant at 2 Hz slow oscillation (i.e., a nonlinear component). (**d**) The masking EMD was then applied to the envelope of the IMF2 of the first layer IMFs (top panel) to produce the second layer IMFs (bottom panel), which contains 4 IMFs. (**e**) The marginal amplitude spectrum of HHT of the second layer IMFs. The strong amplitude of the second IMF in this spectrum were observed at fundamental envelope frequency (2 Hz). The frequency-doubling of envelope was also clearly observed in the spectrum of the first IMF (4 Hz). (**f**) The Holo-Hilbert spectrum of SSVEP response induced by the AM flicker. The amplitude spectrum showed the strong amplitude at 14 Hz carrier frequency, 2 Hz and 4 Hz amplitude modulation in two-dimensional frequency representation. The x-axis represents the carrier frequency (*f*_*c*_), and the y-axis represents the amplitude modulation frequency (*f*_*am*_). At 0.5 Hz y-axis, this x-axis is the summed amplitude of carrier frequencies over time. The frequency axes represent in a dyadic frequency scale. Note that the display of carrier frequencies at 0.5 Hz on the y-axis does not affect the observation of envelope responses since the frequency interest for the envelope is 2 Hz and its higher frequencies. Moreover, this selection for displaying the carrier frequencies at 0.5 Hz y-axis is independent of the lower value (i.e., 0.5 Hz) of the band-pass filter applied to the continuous EEG data.

To examine how the visual system processes the envelope, we manipulated an AM flicker to elicit the SSVEP responses. We also performed a sinusoidal flicker to establish control conditions with no envelope component for HHS comparisons with AM flicker condition (as shown in Fig. 1 and Table 1). To simplify the coordinate expression of the HHSA results for the AM flicker condition, the symbol < *n f*_*am*_ | *m f*_*c*_ > denoting the amplitude modulation frequency (*f*_*am*_) at *n* Hz residing at the *m* Hz carrier frequency (*f*_*c*_) observed in HHS was used for further description. First, in experiment 1, photodiode recordings were used to establish the fundamental differences between Fourier spectrum and HHS for each condition. The observed amplitude spectrum of photodiode signals for sinusoidal and AM flicker conditions was dominated by strong fundamental frequency components (Fig. 2). The Fourier spectrum of AM flicker can only present the additive frequencies (i.e., *f*_1_ = 13 Hz and *f*_2_ = 15 Hz) that do not accommodate the physically meaningful representation that matches the intuitive representation of an AM signal (Fig. 2e). That is, the physical presentation of 14 Hz carrier frequency and 2 Hz envelope could not be obtained by FFT. In contrast, HHSA clearly shows both carrier and envelope frequency of the AM waveform separately in the two-dimensional frequency spectrum (Fig. 2f). The visual evoked responses have been found to be dominantly distributed in the occipital lobe, specifically in the Oz channel. FFT and HHSA were thus applied to calculate the amplitude spectrum of SSVEPs elicited by sinusoidal and AM flicker from the binocular stimulation at Oz channel for further comparisons. To illustrate how SSVEP were analyzed by HHSA in the level of a single subject, the two layer intrinsic mode functions of SSVEP induced by sinusoidal (Fig. 3) and AM (Fig. 4) flicker from binocular stimulation were shown. In the AM flicker condition, the grand average SSVEP response showed a strong 2 Hz amplitude modulation in both time-domain and time-frequency domain (Supplementary Fig. S4).

**Table 1:** The conditions in Experiment 1.

Eye stimulation	Conditions					
	1	2	3	4	5	6
Dominant eye	14 S^a^	Blank	14 S^a^	Blank	(2:14) AM^b^	(2:14) AM^b^
Non-dominant eye	Blank	14 S^a^	14 S^a^	(2:14) AM^b^	Blank	(2:14) AMb

^a^sinusoidal flicker with 14 Hz.

^b^amplitude-modulated flicker with 2 Hz envelope and 14 Hz carrier.

#### The amplitude spectrum of SSVEP responses: FFT and HHSA results

**Figure 5:**
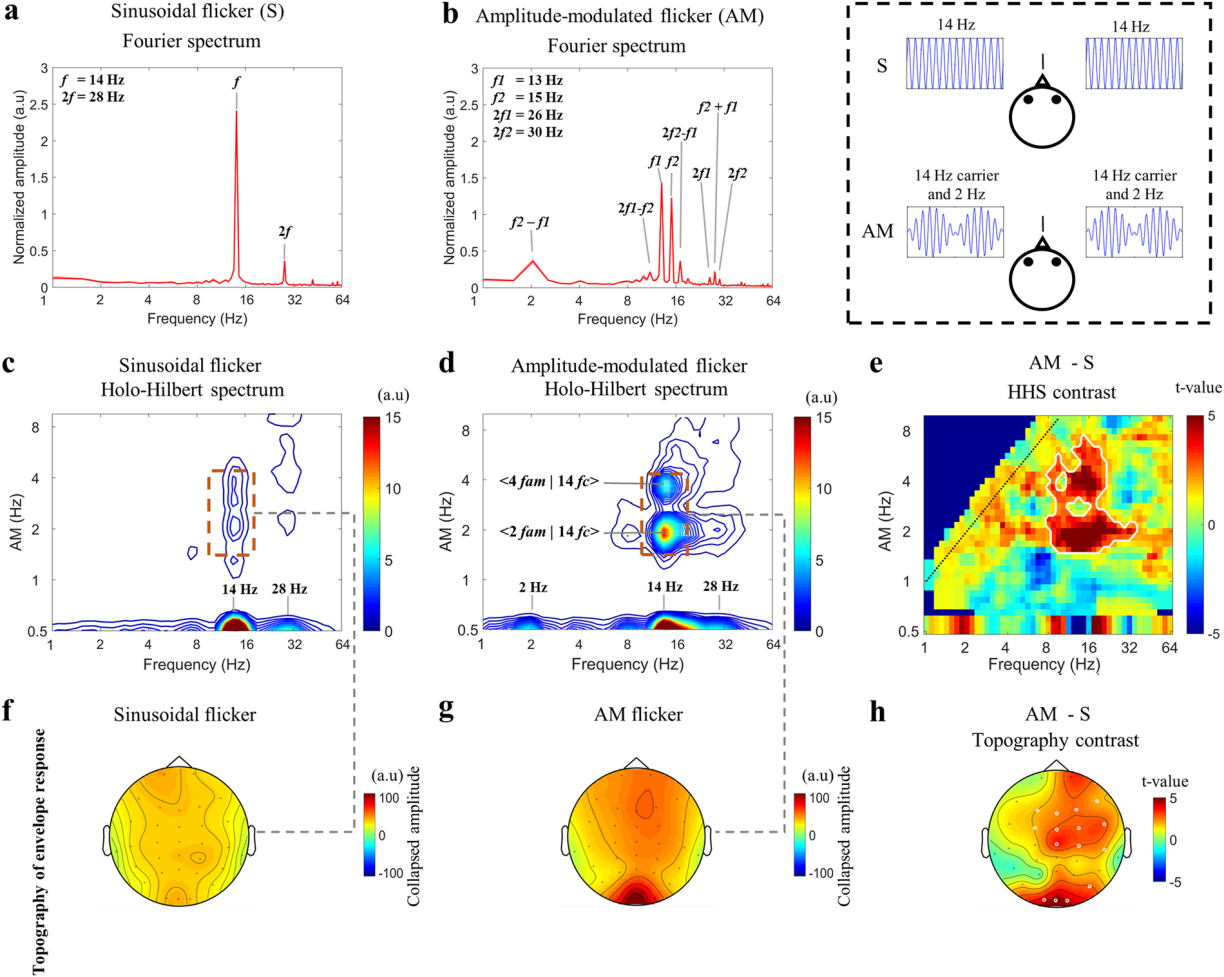
(Experiment 1) The SSVEP amplitudes of AM and sinusoidal flicker at the Oz channel for binocular stimulation, averaged across 13 subjects. The SSVEP amplitudes are observed in the one-dimensional frequency spectrum of FFT analysis and two-dimensional frequency representation of HHSA. The dashed-line panel in the top right corner indicates graphically how the visual stimuli were provided to the participant. (**a**) The SSVEP amplitudes observed in FFT during the sinusoidal flicker condition. (**b**) The SSVEP components observed in FFT during the AM flicker condition. In each subfigure in (**a**,**b**) the x-axis represents the frequency; the y-axis is normalized amplitude spectrum with an arbitrary unit calculated by FFT of the normalized SSVEP response. (**c**) The SSVEP components revealed by HHS in the sinusoidal flicker condition. (**d**) The SSVEP components observed with HHS in the AM flicker condition. In each subfigure in (**c**,**d**) the x-axis represents the carrier frequencies (fc), and the y-axis represents the amplitude modulation (fam). The frequency axes are represented in dyadic frequency. At 0.5 Hz y-axis, the x-axis is the summed amplitude of carrier frequencies over time. (**e**) The HHS contrast of SSVEP responses between AM and sinusoidal flicker. The red area within the white contour indicates areas with significant t-values (p<0.05, df = 12, two-tailed, CBnPP test) of the contrast. A dashed diagonal line at the top left corner indicates the boundary between the fc and fam (fam < fc). (**f**) The HHS topography of the envelope response during sinusoidal flicker shows SSVEP distribution of the collapsed amplitude within the dashed-line rectangle given in (**c**). (**g**) The HHS topography of the envelope response during AM flicker shows SSVEP distribution of the collapsed amplitude within the dashed-line rectangle given in (**d**). (**h**) The significant differences between sinusoidal and AM flicker are represented with white circles in the EEG channel. Each white circle denotes p < 0.05 in the cluster-based non-parametric permutation (CBnPP test) for the electrode.

Figure 5 shows the amplitude spectrum of SSVEPs obtained by FFT and HHSA for sinusoidal and AM flicker conditions at the Oz channel during binocular stimulation. As presented in Fig. 5a,b, the Fourier spectrums showed that both flickers induced SSVEP responses at the fundamental and ambiguously nonlinear components because of nonlinear processes in the visual system. In sinusoidal flicker conditions, SSVEP amplitudes showed peaks at the fundamental frequency *f* and nonlinear components at frequency-doubling 2 *f* (*f* = 14 Hz) (Fig. 5a). In AM flicker conditions, SSVEP spectrums showed a series of nonlinear intermodulation components, in which frequencies could be expressed by the equation: m*f_1_ ± n*f_2_, where *f*_1_ (13 Hz) and *f*_2_ (15 Hz) were the fundamental frequencies, with m and n representing positive integers (Fig. 5b). The spectral peak at 2 Hz (i.e., *f*_2_-*f*_1_) was the slow additive frequency and physically did not correspond to a 2 Hz envelope frequency. Therefore, the neural processing of the envelope could not be fully revealed with Fourier analysis. Similar to single-dimensional frequency representation of Fourier transform, Bispectrum (higher order spectral analysis) also cannot address the effects of the amplitude modulation, which is important to quantify the nonlinearity of envelope response (Supplementary Fig. S5).

As illustrated in Fig. 5c,d, the Holo-Hilbert spectrum showed the carrier and amplitude modulation frequencies of the SSVEP response for each condition in a two-dimensional frequency representation. In the sinusoidal flicker condition, HHS showed strong amplitudes at the fundamental carrier frequency (14 Hz) and its frequency-doubling (28 Hz) (Fig. 5c). Some weak amplitude of 2 to 4 Hz modulating 14 Hz were observed in the sinusoidal condition (within the dashed-line red rectangle in Fig. 5c). Next, we examined the amplitude spectrum of SSVEPs elicited by AM flicker in HHS. Figure 5d shows the SSVEP spectrum at < 2 *f*_*am*_ | 14 *f*_*c*_ > during binocular stimulation analyzed using HHSA physically corresponds to the 2 Hz envelope of the AM flicker. This information cannot be seen with FFT methods given in Fig. 5b. This pattern of results of < 2 *f*_*am*_ |14 *f*_*c*_ > was termed the “fundamental frequency of the envelope”. Interestingly, a < 4 *f*_*am*_ | 14 *f*_*c*_ > evoked spectrum corresponding to the “frequency-doubling of the envelope” was also observed as a rather unexpected nonlinear component, which was not observed in HHS of the photodiode signal (Fig. 2f). Presumably < 4 *f*_*am*_ | 14 *f*_*c*_ > is a byproduct of visual nonlinear dynamics. In addition, the amplitude of carrier frequencies at 2 Hz and 28 Hz were also observed in HHS (the fundamental frequency = 14 Hz, at the 0.5 Hz y-axis) as nonlinear carrier components. Likewise, the fundamental frequencies and nonlinear components of SSVEPs in monocular stimulation were also observed with smaller amplitudes in the Fourier spectrum and HHS compared to binocular stimulation (Supplementary Fig. S6).

To confirm that the < 2 *f*_*am*_ | 14 *f*_*c*_ > and < 4 *f*_*am*_ | 14 *f*_*c*_ > of binocular stimulation observed in HHS were due to the envelope response of AM flicker, cluster-based non-parametric permutation tests (CBnPP^33^) between the SSVEP spectrum of AM and sinusoidal flicker were performed. The 2 Hz *f*_*am*_ modulating wide carrier bands around the fundamental frequency (14 Hz) were significantly different (*n* = 13, *p* < 0.05, *df* = 12, two-tailed, CBnPP t-test, red areas within white contour show the significant t-value) (Fig. 5e). The 4 Hz *f*_*am*_ modulating narrow carrier band was also significantly different. In contrast, the evoked amplitude of monocular stimulation at < 4 *f*_*am*_ | 14 *f*_*c*_ > observed in HHS showed no significant difference between AM and sinusoidal flicker (Supplementary Fig. S7a,b). In addition, these evoked amplitudes at < 2 *f*_*am*_ | 14 *f*_*c*_ > and < 4 *f*_*am*_ | 14 *f*_*c*_ > were also enhanced in binocular stimulation compared to averaging the monocular eye (*n* = 13, *p* < 0.05, *df* = 12, two-tailed, CBnPP t-test, Supplementary Fig. S7c).

#### Occipital activity induced by the envelope of AM flicker

To examine the SSVEP distribution of envelope-induced responses during AM flicker, HHSA was performed on the 32-channel EEG signals. For each channel, we collapsed the SSVEP amplitudes with *f*_*am*_ between 1.5 and 4.5 Hz, and *f*_*c*_ from 12 to 16 Hz obtained with HHSA to show the HHS topographies. In the sinusoidal flicker condition, the HHS topography did not show a clear distribution of an envelope-induced response, presumably due to sinusoidal flicker not inducing much envelope response (Fig. 5f). In contrast, the HHS topography for AM flicker showed very robust SSVEP responses to the envelope, distributed in the occipital channels (Fig. 5g). The comparison between sinusoidal and AM flicker in envelop-induced responses showed that many electrodes carried these responses. These are shown in Fig. 5h, with white highlighted circles denoting the envelope responses for AM flicker that were significantly stronger than those for sinusoidal flicker, principally in the occipital lobe and the anterior channels in binocular stimulation (*n* = 13, p < 0.05, *df* = 12, two-tailed CBnPP test). In contrast, monocular flicker stimulation only exhibited significant increased SSVEP envelope responses in three occipital channels (*p* < 0.05, *df* = 12, two-tailed CBnPP test, Supplementary Fig. S8).

### Experiment 2: Dichoptic stimulation

#### The SSVEP amplitudes showed negative correlation in dichoptic stimulation

**Figure 6:**
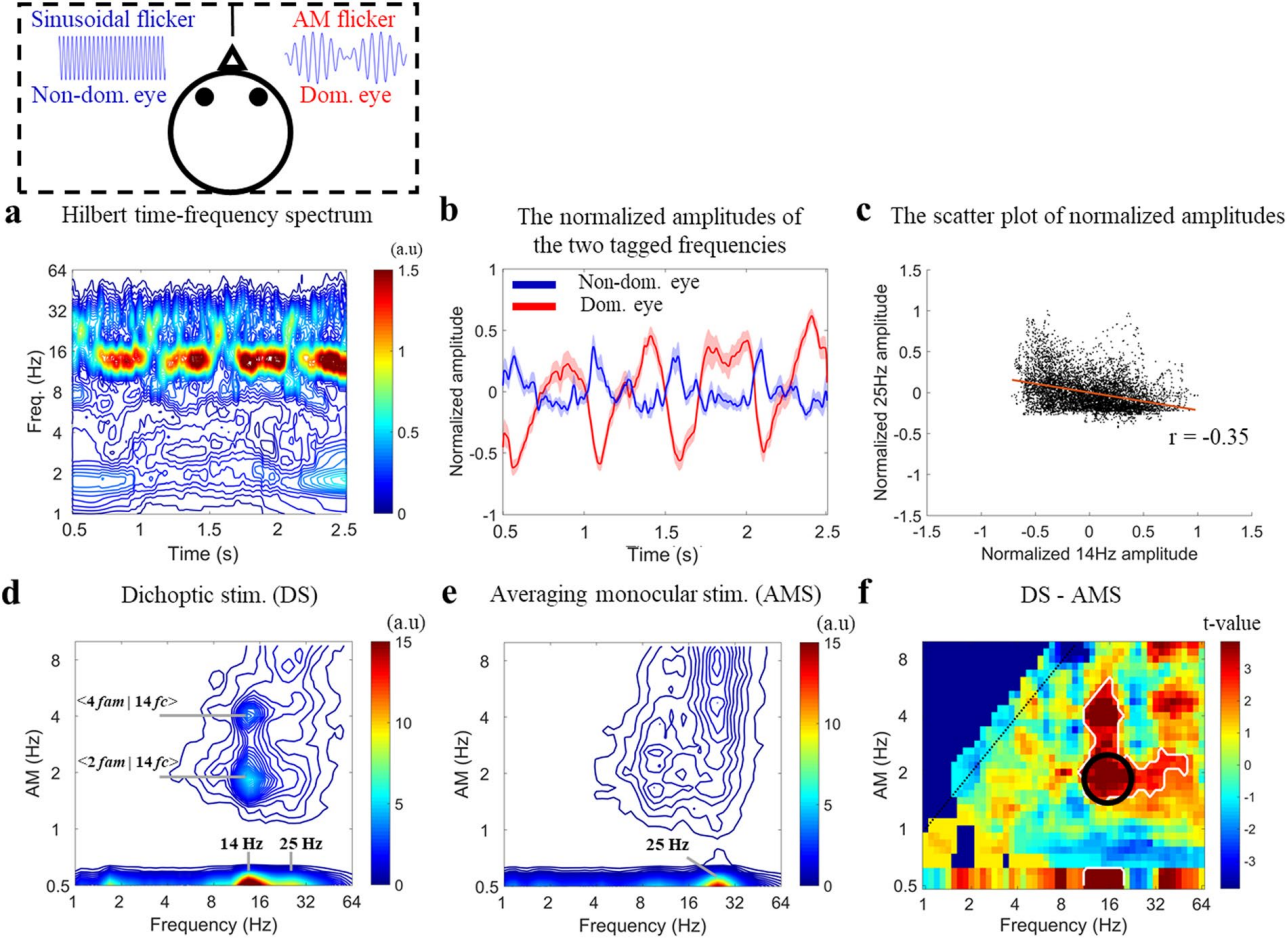
(Experiment 2) The interocular correlation of SSVEP responses across fourteen subjects during dichoptic stimulation. In the top left of Fig. 6, the dashed-line panel illustrates what the visual stimuli were: 2 Hz modulating 14 Hz AM flicker was presented to the dominant eye and 25 Hz sinusoidal flicker was presented to the non-dominant eye simultaneously. (**a**) The spectral amplitude of SSVEPs during dichoptic stimulation were observed in Hilbert-Huang transform spectrum, averaged across subjects for the Oz channel. (**b**) The time course of the amplitude of two eyes’ tagged frequencies from the HHT spectrum. The blue curve shows the time course of amplitude corresponding to the sinusoidal flicker (at 25 Hz) and the red curve shows the time course of amplitude corresponding to the AM flicker (at 14 Hz). Red and blue shaded areas are standard errors of the mean. (**c**) The scatter plot of the amplitudes of the two tagged frequencies showing the strong negative correlation between the two eyes. Each dot corresponds to a 4 ms time bin for the amplitude time course. Data were pooled together across subjects. (**d**) The HHS of SSVEP response during dichoptic stimulation. (**e**) The HHS was calculated by averaging the HHS from each monocular stimulation (including left and right eye). (**f**) The contrast between the HHS from binocular viewing and the mean HHS from each monocular stimulation, showing a significant t-value at < 2 *f*_*am*_ | 14 *f*_*c*_ > (within the dark circle), <4 *f*_*am*_ | 14 *f*_*c*_ > and < 2 *f*_*am*_ | 25 *f*_*c*_ > (white contoured area centered at the red area, *p* < 0.05, *df* = 13, two-tailed, CBnPP test).

To further explore effects of the envelope, we analyzed the SSVEP responses during dichoptic stimulation (as shown in Table 2), in which a AM flicker with 2 Hz envelope and 14 Hz carrier was presented to one eye (i.e., dominant eye) and a 25 Hz sinusoidal flicker was presented to the other eye (i.e., non-dominant eye). First, the amplitude spectra of SSVEPs during dichoptic stimulation were obtained by Hilbert-Huang transforms (HHT)^34^ to show the time-frequency spectrum. This HHT spectrum showed the amplitude modulated 14 Hz signal corresponding to the physically meaningful representation of AM flicker. Likewise, this HHT spectrum also showed an amplitude increase at 25 Hz elicited by sinusoidal flicker. Moreover, the SSVEP amplitude at 25 Hz appeared to be negatively correlated with that at 14 Hz (Fig. 6a). We then averaged the SSVEP amplitude at ±3 Hz distance from the stimulus frequencies obtained in the HHT spectrum (i.e., 14 Hz and 25 Hz) and plotted an average of these amplitudes in time-courses across subjects (Fig. 6b). This plot illustrates a clear counterphase relationship of the SSVEP amplitudes in dichoptic stimulation in which SSVEP amplitude showed repetitive cycles of suppression and dominance mainly driven by the 2 Hz amplitude modulated time-course. These amplitudes were pooled together across subjects and presented in a scatter plot. This scatter plot illustrates the strong significant negative correlation between the two eyes’ SSVEP responses (*r* = −0.35, *p* < 0.001) (Fig. 6c). The negative correlation was also observed when the AM flicker was presented to the non-dominant eye (*r* = −0.26, *p* < 0.001) (Supplementary Fig. S9). These results indicate the negative correlation between the evoked responses of AM and sinusoidal flicker is observed regardless of whether the AM stimuli was presented to the dominant or non-dominant eye.

**Table 2:** The conditions in Experiment 2.

Eye stimulation	Conditions					
	1	2	3	4	5	6
Dominant eye	25 S^a^	Blank	(2:14) AM^b^	Blank	25 S^a^	(2:14) AM^b^
Non-dominant eye	Blank	25 S^a^	Blank	(2:14) AM^b^	(2:14) AM^b^	25 S^a^

^a^sinusoidal flicker with 25 Hz.

^b^amplitude-modulated flicker with 2 Hz envelope and 14 Hz carrier.

#### The neural responses elicited by the 2 Hz envelope of AM flicker from one eye modulated neural responses elicited by 25 Hz sinusoidal flicker from the other eye during dichoptic stimulation

From visual inspection, it was observed in the HHT spectrum that the SSVEP amplitude elicited by 25 Hz sinusoidal flicker might have been modulated periodically by the 2 Hz envelope of the AM flicker. However, HHT could not precisely quantify the amplitude modulation frequencies at 25 Hz. Therefore, HHSA were performed to gauge the degree of amplitude modulation frequencies modulating the 25 Hz SSVEP amplitude. First, SSVEPs during dichoptic stimulation were analyzed with HHSA and then were shown in two-dimensional frequency spectrum HHS. In HHS, the SSVEP amplitudes at < 2 *f*_*am*_ | 14 *f*_*c*_ >, < 4 *f*_*am*_ | 14 *f*_*c*_ > and 14 Hz (the fundamental carrier frequency) elicited by AM flicker and 25 Hz elicited by sinusoidal flicker were concurrently observed (Fig. 6d). Next, the SSVEP responses elicited by 25 Hz sinusoidal flicker in the left and right eye stimulation were analyzed by HHSA. The averaged HHS of SSVEP responses from each monocular stimulation showed an amplitude increase at 25 Hz in the two-dimensional frequency spectrum (Fig. 6e). To investigate which amplitude modulation frequencies modulated 25 Hz in dichoptic stimulation compared to averaging monocular stimulation, HHS comparison between the SSVEP spectrum in the two conditions was performed. The HHS comparison revealed a significant difference centered on the 2 Hz *f*_*am*_ modulating 14 Hz *f*_*c*_ (white contoured area centered on the red area within the dark circle in Fig. 6f, n = 14, p < 0.05, df = 13, two tail, CBnPP test) and propagated the significant area to 4 Hz *f*_*am*_ modulating 14 Hz *f*_*c*_ and also 2 Hz *f*_*am*_ modulating 25 Hz *f*_*c*_. In a similar manner, the 25 Hz SSVEP amplitude from the dominant eye was also modulated by 2 Hz (n = 14, p < 0.05, df = 13, two tail, CBnPP test) (Supplementary Fig. S9f). These results indicated that 25 Hz SSVEP amplitude from one eye was only modulated by the 2 Hz envelope of AM flicker from the other eye during dichoptic stimulation regardless of eye dominance.

## Discussion

In the present study, we observed that not only can an evoked response to the envelope at fundamental and doubling frequencies be quantified, but that the interaction between the envelope and carrier is also revealed by HHSA. By applying HHSA and SSVEP together, we can study the human visual system from a nonlinear prospective in terms of high-dimensional frequency representations and also provide additional valuable and unreported information, which is commonly overlooked by traditional methods based on the additive expressions in one-dimensional frequency representations. The justification of the nonlinear view is rather straightforward: the brain processes are nonlinear^2,21,35^.

Although some previous studies have demonstrated that the visual system is nonlinear^11,15,16,18^, in those studies, FFT was mainly used as the analytical method and therefore could not reveal any dynamic interactions apart from those in the one-dimensional frequency spectrum. Consistent with earlier studies^11–13^, the current results showed the intermodulation components and harmonic frequencies observed in FFT of data obtained with AM stimuli as the nonlinear components. It should be noted that the nonlinear component at the 2 Hz slow additive oscillation (i.e. *f*_2_-*f*_1_) observed in FFT did not physically correspond to the time-varying envelope of AM flicker (Fig. 5b). This slow additive oscillation may come from the asymmetrical evoked amplitudes of AM flicker induced by a rectifier, in which neurons would increase their firing rate with increments in the light level and only slightly decrease with a decrement in the light level^36^. AM flicker contains an envelope component and Fourier analysis cannot account for neural responses induced by the envelope. Even though the HHT showed a strongly evoked amplitude at 14 Hz single frequency with amplitude modulation, the HHT could not precisely quantify the frequency characteristics of this amplitude modulation (Supplementary Fig. S4e).

To resolve this issue, HHSA was applied to SSVEP responses induced by AM flicker to elucidate the neural mechanism underlying the nonlinear visual system. As observed, HHSA clearly quantified evoked responses of fundamental envelope frequency at 2 Hz *f*_*am*_ modulating 14 Hz *f*_*c*_ (<2 *f*_*am*_ | 14 *f*_*c*_>) observed in HHS that physically corresponded to the 2 Hz envelope of AM flicker from the photodiode recording. In addition, HHSA was used to discretely calculate the SSVEP amplitude of <2 *f*_*am*_ | 14 *f*_*c*_> (i.e., fundamental envelope frequency), and the 2 Hz slow carrier frequency in the two-dimensional frequency spectrum indicates a possible correlation between neural responses to these frequencies and the beat perception (Fig. 5d). Specifically, the beat perception is visual perception capable of recognizing luminance undulations at a frequency equal to the difference between the two different temporal frequencies^17^. Such beat perception has been reported in many previous studies investigating temporal visual processing^17,37^. However, since FFT only quantified the carrier frequencies without amplitude modulation information, the relationship between beat perception and neural responses was still hard to interpret using FFT analysis. It is because the neural responses correlating with beat perception might not arise only from the 2 Hz carrier component observed in the FFT results, and might originate from the neural response of the 2 Hz amplitude modulation, as observed in Holo-Hilbert spectrum.

Interestingly, the evoked frequency-doubling of the envelope (i.e., 4 Hz *f*_*am*_ modulating 14 Hz *f*_*c*_, <4 *f*_*am*_ | 14 *f*_*c*_ >) was also detected as a nonlinear component which cannot be quantified in one-dimensional frequency representations provided by FFT-like methods. Since this < 4 *f*_*am*_ | 14 *f*_*c*_ > frequency-doubling is not observed in HHS of the AM signal of the photodiode (Fig. 2f), this might have emerged from some additional nonlinear neural mechanisms encoding the temporal envelope of stimuli in the visual system. For example, in mammal studies, the Y cells in the lateral geniculate nucleus and some neurons in area 18 of the cat have been reported to be responsive to the envelope and also generate a frequency doubling of the envelope response^38–40^. Rosenberg & Issa (2011) also described that the physiological circuitry of retinal ganglion Y cells might process the envelope as the demodulating circuit. Such a demodulating mechanism may explain the appearance of frequency-doubling of the envelope. In this mechanism, at the first stage, the SSVEP elicited by AM flicker is band-pass filtered centered on the 14 Hz carrier. Then, the second stage rectifies the output signal of the first-stage filter. In the third stage, the signal passes through a low-pass filter eliminating high frequencies and retains only the low frequency components to produce the envelope frequencies. This mechanism was also consistent with the processing scheme of complex natural scenes, in which second-order properties (i.e., contrast, texture) were processed by the three main stages of filter-rectify-filter cascade^41–43^. The extracted envelope frequencies might be composed of fundamental frequencies and also harmonic frequencies due to the distortion of the envelope waveform during nonlinear processing at the second stage. Consequently, the <4 *f*_*am*_ | 14 *f*_*c*_> frequency doubling of the envelope can be observed as nonlinear components in the current results. Note that the evoked responses at frequency-doubling of the envelope were significantly observed only during the binocular stimulation, not during monocular stimulation. One plausible reason for this could be that the evoked potentials of the envelope at fundamental and doubling frequencies in binocular viewing would have been enhanced when compared to averaging monocular viewing (Supplementary Fig. S7c). This finding was consistent with the previous studies that found the binocular stimulation signals with similar stimuli converged at binocular neurons, evoking a larger amplitude when compared to a monocular stimulation^44,45^. This result indicated that the monocular neural response of the fundamental and double envelope frequencies may partially integrate into binocular cells, which fits with binocular summation models^46,47^.

In addition, since the evoked potentials represent the collective activities of a large population of neurons^35^, SSVEP distribution of envelope responses is important to determine the cortical areas involved in neural activity related to envelope processing. Previous animal studies with unit recordings showed that most complex cells in area 18 were envelope-responsive^8,39–41^. The current findings have also shown that the evoked potentials of the envelope have a strong activity in the occipital lobe suggesting that the neural response to envelopes is probably processed in early human visual cortex.

The current study also found that the 2 Hz *f*_*am*_ did not only modulate the 14 Hz stimulation but also propagated to a wide range of carrier frequencies in both monocular and binocular stimulation. These results indicated that the neural response of AM flicker induced a nonlinear multiplicative modulation between the 2 Hz envelope and adjacent carrier frequencies around the 14 Hz fundamental carrier frequency as a part of nonlinear processing in the visual system. This nonlinear multiplicative modulation observed in HHS may also explain the puzzled observation of intermodulation and harmonic components in FFT analysis in which the peak frequencies of SSVEP response showed a 2 Hz difference between two nearby frequencies in the wide range frequencies (i.e., 9 Hz, 11 Hz, 13 Hz, 15 Hz, 17 Hz, 19 Hz, 26 Hz, 28 Hz, 30 Hz, etc.) (Fig. 5b).

The above findings illustrated how the envelope of the amplitude-modulated stimulus was processed in the visual system during monocular and binocular stimulation. To further understand how the neural responses to the envelope affects the competing signals in dichoptic stimulation we also examined the interaction between a 2 Hz envelope modulating a 14 Hz AM signal presented in one eye and a 25 Hz uniform sine-wave signal presented in the other eye. The SSVEP response to the sinusoidal flicker from one eye presented four repetitive cycles of suppression and dominance in two seconds and further showed a negative correlation with the evoked response to AM flicker from the other eye (Fig. 6 and Supplementary Fig. S9). In humans, interocular inhibition has been reported with psychophysical methods and visual evoked potentials when the two eyes carrying signals that differ from each other^29,48–51^. The current results further demonstrated a detailed and dynamic negative correlation similar to previous investigations in which the binocular rivalry paradigm were applied to investigate the neural correlates of perceptual dominance^52,53^. Brown & Norcia (1997) reported a negative correlation between SSVEP amplitude by using frequency-tagged gratings in a binocular rivalry experiment and showed that perceptual rivalry plays a critical role in changing the SSVEP response to flicker. Moreover, in this study, HHSA revealed that the 25 Hz evoked response from one eye was dominantly modulated by 2 Hz, which was the fundamental envelope frequency of AM flicker from the other eye. Consequently, the neural responses to incoming signals might have been greatly affected by the envelope of the AM flicker and this could explain the negative correlation between the two eyes’ responses. There was also a certain degree of perceptual response components originated from the envelope, thus affecting the evoked response to the competing signal.

In conclusion, a novel method, HHSA, was utilized in the current study to elucidate the neural mechanisms underlying the nonlinear responses of the visual system by analyzing SSVEP responses to AM flicker. The evoked responses to AM flicker observed with HHSA could naturally depict the intertwined characteristics of nonlinear neural responses in the visual system, yielding new insights into how envelope, carrier and their interactions were processed in the human brain. The results not only show the appearance of frequency doubling of the envelope in SSVEP signals likely emerged from nonlinear properties of the visual system, but also indicated nonlinear multiplicative modulation induced by processing of slow multiplicative oscillations (i.e., a 2 Hz envelope). Specifically, the evoked responses of the fundamental envelope frequency at (< 2 *f*_*am*_ | 14 *f*_*c*_ >) observed with HHS may provide additional measures for the correlation between envelope responses and perception. Finally, our findings also highlight the important point that the envelope also plays a critical role in modulation of competing neural responses during dichoptic stimulation. These findings cannot be revealed and clarified in one-dimensional frequency representations obtained with FFT analysis. Therefore, this HHSA study yields new insight into the mechanism of amplitude modulated signal processing in the visual system and also elucidates the nonlinear characteristics of the brain.

## Materials and Methods

### Participants

Thirteen (seven females; mean age = 24.23 years, SD = 3.05 years) healthy students from National Central University of Taiwan participated in experiment 1. Fourteen neurologically healthy students participated in experiment 2 (seven females; mean age = 23.36 years, SD = 3.13 years). Six of the thirteen subjects from experiment 1 participated in experiment 2. All participants had normal or corrected-to-normal vision and were neurologically healthy with no history of psychiatric disorders. Applicants with first-degree relatives with epilepsy or migraine were excluded from the study. This study was carried out in accordance with the Social and Behavioral Research Ethical Principles and Regulations of National Taiwan University with written informed consent from all the participants. All experimental procedures were approved by the Institutional Review Board of National Taiwan University, Taiwan.

### Stimuli and procedures

The stimulus presentation device was composed of two separated black tubes (approximately 13 cm in length) each with one white LED covered with a 4 × 4 diffuser plate at one end of the tube to form a stimulus size of ~18.2° visual angle to each eye. The centers of each tube were 4.5 cm apart from each other. Each LED generated a stimulus of mean luminance of 39.2 cd/m^2^ for the experiments.

A custom-made device was used for generating different light flicker waveforms for the two experiments in this study. The hardware was composed of a 16-bit digital-to-analog converter (NI USB-6229 BNC, National Instruments, Austin, Texas, USA) that drove the LED signal at a rate of 40 kHz. To verify that the emitting signal had the desired shape, an integrated photodiode (BPW34, OSRAM Opto Semiconductors) collected the output signal and recorded it with a BioPac MP35 (Biopac Systems, Inc.)

The visual stimuli consisted of both sinusoidal and amplitude-modulated flicker. The stimuli were generated by using MATLAB in-house programs (The MathWorks Inc., Natick, MA, USA) with the following equations:

1 $$\mathrm{Sine}-\mathrm{wave}:\,S(t)={L}_{0}+{L}_{0}\,\sin (2\pi {f}_{c}T)$$

2 $${\rm{AM}}\,\mathrm{waveform}:\,AM(t)={L}_{0}+{L}_{0}\,\sin (2\pi {f}_{m}T)\,\sin (2\pi {f}_{c}T)$$

Where T was a duration of sinusoidal and AM flicker, L_0_ was the mean of the luminance, *f*_*c*_ was the carrier frequency, and *f*_*m*_ was the modulation frequency.

In experiment 1, a 14 Hz single frequency sinusoidal flicker without any envelope was used as a control condition with 30 trials for baseline comparisons (see Eq. 1). The AM flicker was generated by a product of two sinusoidal signals, in which one sinusoidal signal was a fast-changing oscillation (i.e., the carrier frequency, *f*_*c*_ = 14 Hz) and the other sinusoidal signal was a slow-changing oscillation (i.e., the modulation frequency, *f*_*m*_ = 1 Hz) (see Eq. 2). Note that the envelope of this AM flicker was twice the modulation frequency (i.e., *f*_*env*_ = 2 Hz). Mathematically, however, this AM flicker could also be treated as the superposition of two sinusoidal waveforms (i.e., *f*_1_ = 13 and *f*_2_ = 15 Hz) with equal amplitudes. To acquire reliable SSVEP elicited by this complex waveform (AM flicker), we increased the trial number of AM condition to be 50. Therefore, there was a total 240 trials with six conditions (as shown in Table 1) included in experiment 1 to explore the nonlinear characteristics of SSVEPs induced by monocular stimulation (dominant and non-dominant eye, measured with the Miles Test^54^) or binocular stimulation (i.e., two eyes were presented with the same flicker).

To further detect the role of the envelope in dichoptic stimulation, the visual stimuli in experiment 2 consisted of concurrent presentation of a 25 Hz sinusoidal flicker and an amplitude-modulated flicker with 2 Hz envelope and 14 Hz carrier. Experiment 2 also contained six conditions with 20 trials in each condition (Table 2). Trials of the six conditions in each experiment were presented in a randomized order and divided into four blocks, with blocks separated by a short break.

The details of the procedure in experiment 1 and experiment 2 were the same. Participants were asked to press any key to initiate the first trial. After the keypress, participants heard a beep sound indicating they should open their eyes and fix their sight on the black point of the diffuser plate LED for 2.5 s. This was followed by a 2-second break in which the participants were instructed to take a rest where they could blink or close their eyes (Fig. 1). After completion of a trial, another beep occurred to indicate the start of a new trial.

### EEG data acquisition and preprocessing

EEG activity was recorded using an elastic cap (Electrocap International) containing 36 Ag/AgCl electrodes arranged according to the International 10–20 system. The EEG signal was re-referenced to the average of the right and left mastoids. A ground electrode was set between the FPz and Fz electrodes. A Neuroscan amplifier (Nuamps) and Neuroscan 4.2 software were used to record the data with a sampling rate of 1000 Hz. The impedance for every electrode was kept below 5 kΩ during the recordings.

The recorded EEG data were first preprocessed by a 0.5–50 Hz band-pass filter. The data were epoched from 0 to 3000 ms relative to stimulus onset for each trial. The epoched data were detrended before excluding trials with blinks or other artifacts (exceeding 100 uV) from further analysis. Then, the artifact-free data were averaged across trials in each condition to obtain the SSVEP. The SSVEPs were normalized by dividing the standard deviation across EEG channels and conditions for each subject. After these preprocessing procedures, the normalized SSVEP responses from the Oz channel were used as the main source of evoked response for further data analysis^55–57^. To quantify the spectral amplitude in each condition, we used the normalized SSVEP windows from 500 to 2500 ms after the onset of each stimulus to exclude the VEP and to increase the signal to noise ratio of the SSVEP^58,59^.

### Steady state visually evoked potential analysis

In each experiment, the normalized SSVEP elicited by each condition from 0.5 to 2.5 s after onset was analyzed with Holo-Hilbert spectral analysis to obtain a two-dimensional frequency spectrum, in which one dimension was carrier frequencies (*f*_*c*_), and the other dimension was amplitude modulation frequencies (*f*_*am*_). Both axes are represented in dyadic frequency scales, in which the resolution is eight log2 scale bins (e.g., [8 16] contains eight-frequency bins). The contour represents the energy density. More details of HHSA are provided in Supplementary Materials and Methods.

In addition, the amplitude spectrum of SSVEP responses in experiment 1 was also computed with fast Fourier Transform (FFT) by applying the “*fft”* function in Matlab to compare with the results from HHSA. In experiment 2, to investigate the temporal correlation of SSVEP responses between the two eyes, Hilbert-Huang transform^34^ was performed on the normalized SSVEP responses for each condition. The amplitude responses observed in this time-frequency spectrum were then averaged at ±3 Hz distance from the stimulus-frequency (i.e., 11–17 Hz for 14 Hz and 22–28 Hz for 25 Hz for SSVEP responses elicited by AM and sinusoidal flicker, respectively) to extract the amplitudes corresponding to the stimulus frequency. These extracted SSVEP amplitudes were then examined by calculating the Pearson-r correlation coefficients and p-values for the pooled data of all subjects, yielding one coefficient.

### Statistical analysis

Cluster-based non-parametric permutation (CBnPP) analysis was used for evaluation of the spectral differences in HHS. CBnPP is an optimal way for conducting multiple-comparisons while retaining reasonable statistical power. It identifies clusters of significant differences between conditions in space, time and frequency dimensions, making it possible to calculate the natural analysis of interactions between time points, electrodes, and frequency bins^33^.

First, for comparison of every data point (i.e. amplitude spectra of HHSA) between two conditions, a simple dependent-samples t-test was performed to give uncorrected p-values. Adjacent data points exceeding a 5% preset significance level were grouped into clusters. The t statistics for each cluster were summed together for further use in a cluster-level test statistic. A null distribution assuming no difference between conditions was then created. Distributions were generated by randomly assigning the conditions in subjects 2000 times and calculating each randomization’s largest cluster-level statistic. Finally, the cluster-level test statistics and null distribution were compared against each other. Clusters in the highest or lowest 2.5^th^ percentile were considered significant.

SSVEP multichannel amplitude responses from two conditions were then contrasted with each other. Two EEG sensors were defined as neighbors if their distance was no more than 60 mm from each other. We then proceeded with 2000 permutations for each test.

## Supplementary information

Supplementary Information

## Data Availability

The data that support the findings of this study will be available from the corresponding author upon reasonable request.
