# Supplementary material for: Unraveling nonlinear electrophysiologic processes in the human visual system with full dimension spectral analysis
Source: Sci Rep. 2019 Nov 15;9:16919. doi: 10.1038/s41598-019-53286-z (PMC6858326; doi:10.1038/s41598-019-53286-z)
Supplement: Supplementary file 1 — Supplementary Information [file 41598_2019_53286_MOESM1_ESM.docx]

Supplementary Information

**Unraveling nonlinear electrophysiologic processes in the human visual system with full dimension spectral analysis**

**Kien Trong Nguyen^1^_,_ Wei-Kuang Liang^1,2^, Victor Lee^1^, Wen-Sheng Chang^1^, Neil G. Muggleton^1,2,3,4^, Jia-Rong Yeh^2,5^, Norden E. Huang^2,5,6^, Chi-Hung Juan^1, 2^***

^1^ Institute of Cognitive Neuroscience, National Central University, Taoyuan, Taiwan;

^2^ Brain Research Center, National Central University, Taoyuan, Taiwan;

^3^ Institute of Cognitive Neuroscience, University College London, London, UK_;_

^4^ Department of Psychology, Goldsmiths, University of London, London, UK;

^5^ Data Analysis and Application Laboratory, The First Institute of Oceanography, Qingdao, China;

^6^ Pilot National Laboratory of Marine Science and Technology, Qingdao, China.

*** Corresponding author:**

**Chi-Hung Juan**

Email: [chijuan@cc.ncu.edu.tw](mailto:chijuan@cc.ncu.edu.tw)

**Supplementary Materials and Methods**

**Holo-Hilbert spectral analysis.**

Holo-Hilbert spectral analysis consists of two-layer empirical mode decomposition (EMD). The first layer EMD provides the first layer intrinsic mode functions (IMFs), and the second layer EMD provides the second layer IMFs, which contains the IMFs of each envelope functions from first layer IMFs (see the illustration of two-layer EMD in **Supplementary Fig. S1**). Hilbert transform is applied to this two-layer EMD to estimate the instantaneous frequency and amplitude (for details, please see Huang et al.’s study ^1^). Because the two-layer EMD employs a second layer decomposition on the envelope of each IMF, it requires that all the given IMFs are free from the mode-mixing problem. To better resolve this problem that might potentially distort the results of HHSA, in this study we employed an enhanced algorithm of masking EMD proposed by Tsai et al.’s study ^2^. This improved algorithm is modified from the original masking EMD ^3^ to obtain each IMF.

Mathematically, the data analysis proceeds as follows:

Estimate the instantaneous frequency and amplitude of IMFs with Hilbert Transform:

1. Extract a set of the first layer IMFs with EMD from the signal, then estimate instantaneous frequencies and amplitudes of IMFs using Hilbert Transform. These first layer IMFs are known as the first layer EMD of HHSA. This step generates the Hilbert time-frequency spectrum of first layer IMFs and it is named HHT.
2. Construct the envelopes on the first layer IMFs as defined by three studies ^1,4,5^.

- Obtain the absolute value of the IMFs.
- Identify all the absolute-valued function of IMF maxima.
- Assemble the envelope by employing a natural spline through all the maxima.

1. Perform masking EMD on each extracted envelope of each first layer IMF to get the second layer IMFs. Extract instantaneous amplitude modulation frequency (*f_am_*) for the second layer IMFs.
2. Project the second layer EMD result to (*f_am_*, *f_c_*, time) space to form the three-dimensional Holo-Hilbert Spectrum which contains a complete description of the within and cross-frequency dynamics within the time-series ^1^.
3. To aid interpretability, we sum the spectral power (amplitude^2^) marginally over the time dimension to generate the two-dimension HHS, in which the y-axis represents *f_am_*, and the x-axis shows *f_c_*. The square root of those summed power spectrum from the SSVEP results are further calculated to aid visualization.

### Bispectrum analysis

Bispectral analyses were carried out using modified functions from the higher order spectral analysis (HOSA) toolbox ^6^. The Bispectrum was estimated for all possible frequency pairs (*f_1_, f_2_*) using the direct (FFT-based) approach with an FFT length of 1024 with seven sample frequency domain smoothing. The first quarter of the spectrum was extracted from the [2048 x 2048] Bispectrum matrix and used for further analyses.

**Nonlinear mode decomposition**

The nonlinear mode decomposition is a time-frequency based method to decompose a signal into different nonlinear modes ^7^. In this current study, the NMD toolbox v2.0 and customized MatLab codes (The MathWorks Inc., Natick, MA, USA) were used to perform the analysis in simulation. To decompose the signal into a set of nonlinear modes, the ‘*nmd(sig,fs,’ModeNum’,11)*’ function in NMD toolbox was utilized with the sampling rate fs equal to 1000.

**Swarm decomposition**

The Swarm decomposition, mainly based on swarm filtering, is a novel approach to decompose the non-stationary signal ^8^. In this current study, to decompose the signal into a set of mono-component signals, the ‘*SwD*’ function (The available code was shared on website https://github.com/gkaposto/Swarm-Decomposition) and customized MatLab codes (The MathWorks Inc., Natick, MA, USA) were used with the sampling rate of 200 and the following SwD parameters:

- P_th = 0.1
- StD_th = 0.1
- Welch_window = 100 (length of data divided by 8)
- Welch_no_overlap = 50 (welch_window divided by 2)

**Supplementary Figures**

| *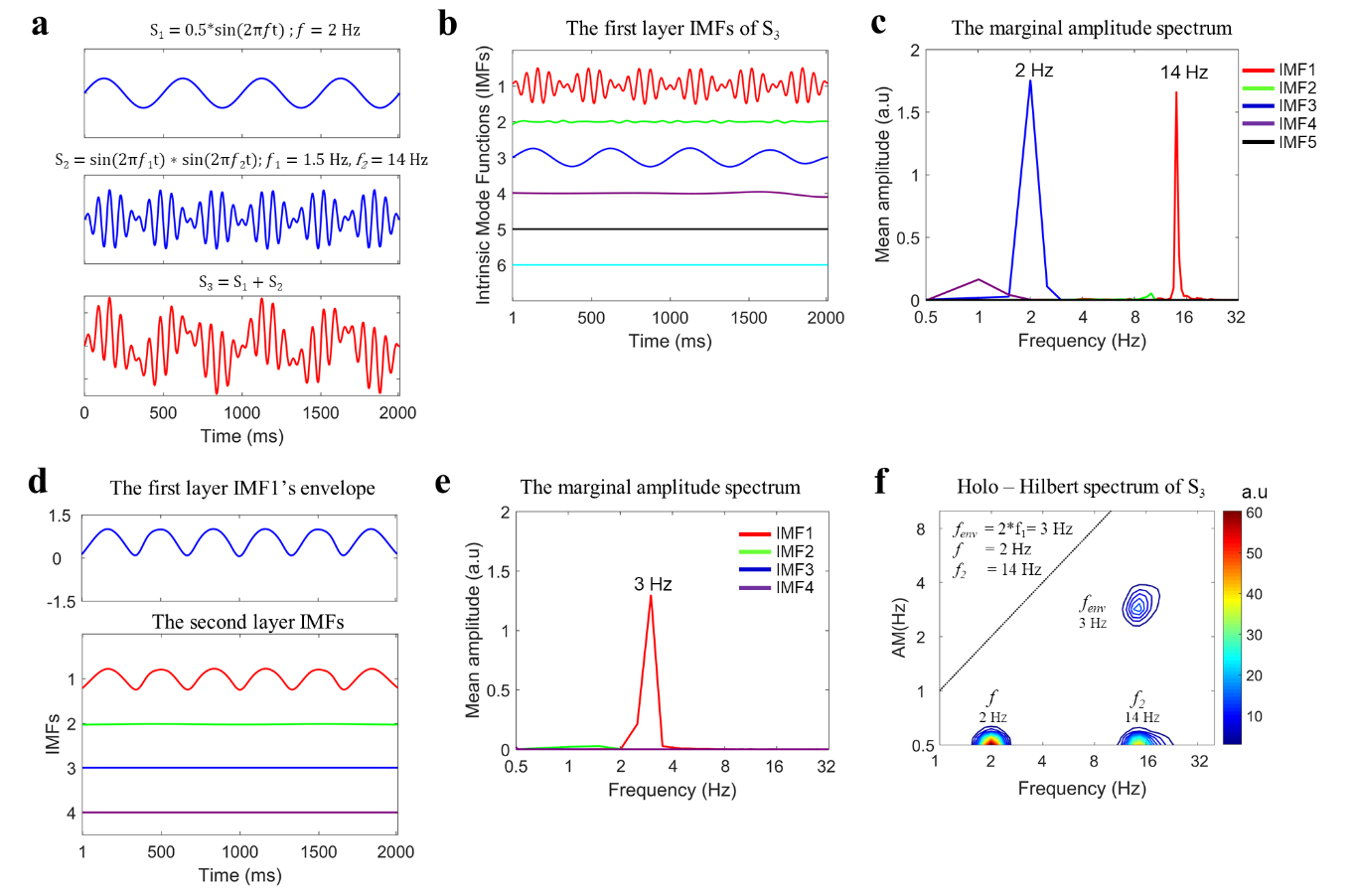* |
| --- |
| **Supplementary Figure S1**. Illustration of two layer IMFs from a simulation signal. **(a)** The signal S_1_ was constructed from a sinusoid with frequency 2 Hz and amplitude 0.5 (top panel). The signal S_2_ was generated by a product of two sinusoidal signals, in which the fast-changing oscillation was the carrier frequency (i.e., *f_2_* = 14 Hz) and the slow-changing oscillation was the modulation frequency (i.e., *f_1_* = 1.5 Hz) (middle panel). Note that the envelope of this AM flicker is twice the modulation frequency (i.e., *f_env_* = 2**f_1_* = 3 Hz). The signal S_3_ was designed as an additive expansion (i.e., S_3_ = S_1_ + S_2_) to have a 2 Hz slow oscillation and a 3 Hz envelope modulating a 14 Hz carrier (bottom panel). **(b)** The first layer IMFs of S_3_ contains six intrinsic mode functions (IMFs). Clearly, counting from the top, the first IMF corresponds to S2, and the third IMF corresponds to S1. **(c)** The marginal amplitude spectrum of Hilbert-Huang transform of the first layer IMFs. The strong amplitudes of first and third IMF in this spectrum were observed at 14 Hz and 2 Hz, respectively. **(d)** The masking EMD was then applied to the envelope of the first IMF (IMF1; 3Hz, top panel) to produce the second layer IMFs (bottom panel). The clear pattern of the 3 Hz modulating sinusoidal wave can be seen in the first IMF (counting from the top) corresponding to the envelop given in S_2_. **(e)** The marginal amplitude spectrum of Hilbert-Huang transform of the second layer IMFs. The strong amplitude of first IMF in this spectrum was observed at 3 Hz. **(f)** The amplitude of carrier and amplitude modulation of S_3_ are represented in the two-dimensional Holo-Hilbert spectrum. The x-axis represents the carrier frequency (*f_c_*), and the y-axis represents the amplitude modulation frequency (*f_am_*). At 0.5 Hz y-axis, this x-axis is the summed amplitude of carrier frequencies over time. The frequency axes represent in dyadic frequency. This simulation analysis demonstrated that the HHS could successfully separate the 2 Hz slow additive oscillation, the 3 Hz slow multiplicative oscillation (i.e., the envelope) and the 14 Hz fast oscillation embedded in signal S_3_. |

| 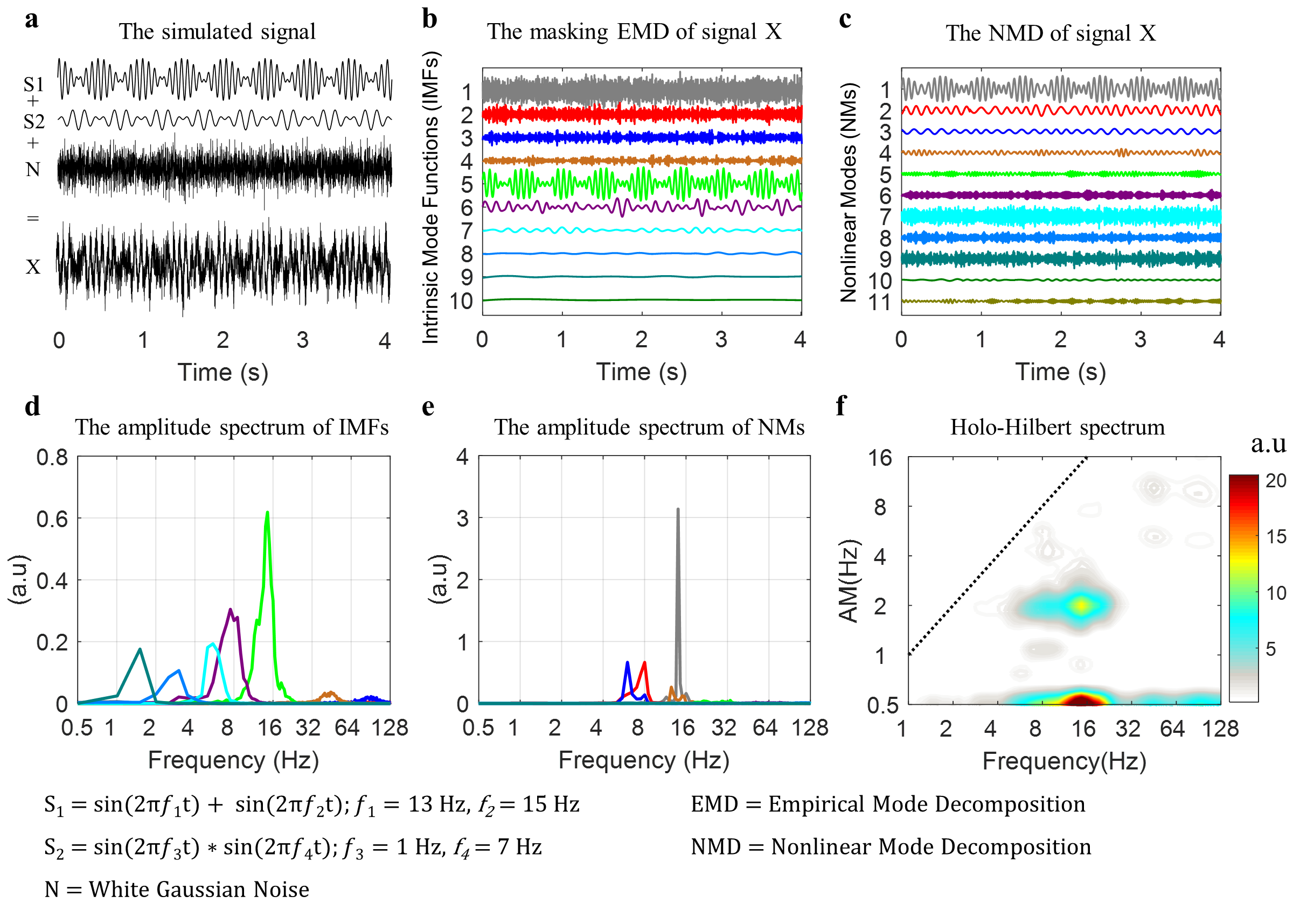 |
| --- |
| **Supplementary Figure S2**. Illustration of intrinsic mode functions and nonlinear modes from a simulation signal. **(a)** The signal S1 was generated by a sum of two sinusoidal signals with frequency of 13 Hz and 15 Hz (top panel). The signal S2 was constructed by a product of two sinusoidal signals, characterized by the modulation frequency (1 Hz) carrier frequency (7 Hz) (middle panel). The signal N was white Gaussian noise. The signal X was a sum of three signals (i.e., X = S1 + S2 + N) (bottom panel). **(b)** The masking EMD decomposed signal X into ten IMFs. Clearly, counting from the top, the fifth IMF corresponds to S1, and the sixth IMF corresponds to S2, retaining the physical meaning in the decomposed components (i.e., IMFs). **(c**) Eleven nonlinear modes were decomposed by the Nonlinear Mode Decomposition. Counting from the top, the first nonlinear mode corresponds to the signal S1 while the second and third nonlinear modes correspond to two sinusoidal components with frequency of 6 Hz and 8 Hz, respectively. **(d)** The marginal amplitude spectrum of ten IMFs. **(e)** The marginal amplitude spectrum of eleven NMs. In the subfigures **2d** and **2e**, the color lines are consistent with the subfigure **2b** and **2c**, respectively. (f) the Holo-Hilbert spectrum of signal X. |

| 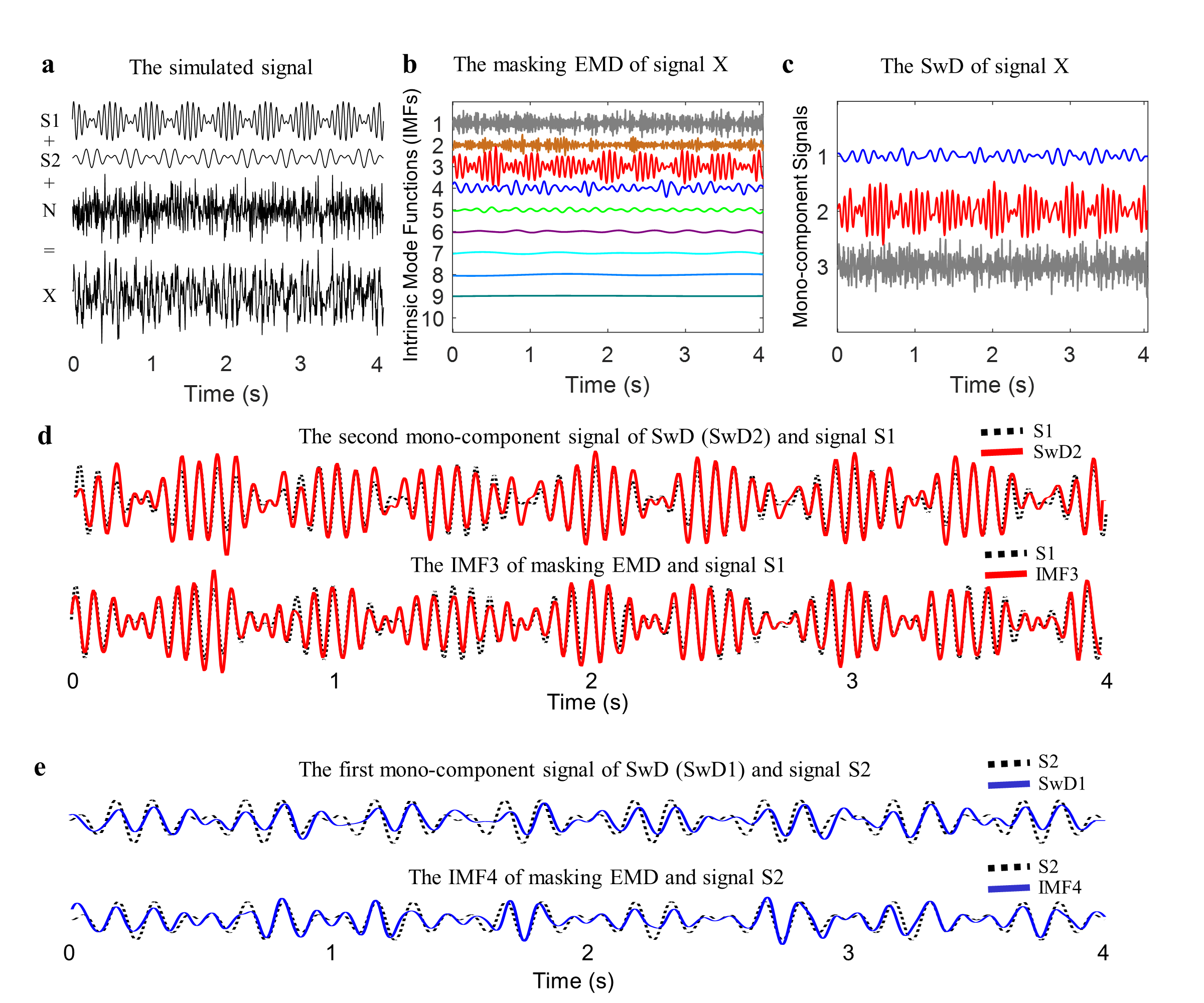 |
| --- |
| **Supplementary Figure S3**. Comparison between masking EMD and Swarm decomposition from a simulation signal. **(a)** The same simulation signal, as shown in **Supplementary Figure S2,** with sampling rate of 200 Hz. **(b)** The masking EMD decomposed signal X into nine IMFs. Clearly, counting from the top, the third IMF corresponds to S1, and the fourth IMF corresponds to S2, retaining the physical meaning in the decomposed components (i.e., IMFs). **(c**) Three mono-component signals were decomposed by the Swarm Decomposition. Counting from the top, the first mono-component signal corresponds to the signal S2 while the second mono-component signal correspond to the signal S1. **(d)** The signal S1 and decomposed components of SwD (top panel) and masking EMD (bottom panel). **(e)** The signal S2 and decomposed components of SwD (top panel) and masking EMD (bottom panel). In the subfigures **3d** and **3e**, the color lines are consistent with the subfigure **3b** and **3c**, respectively. |

| 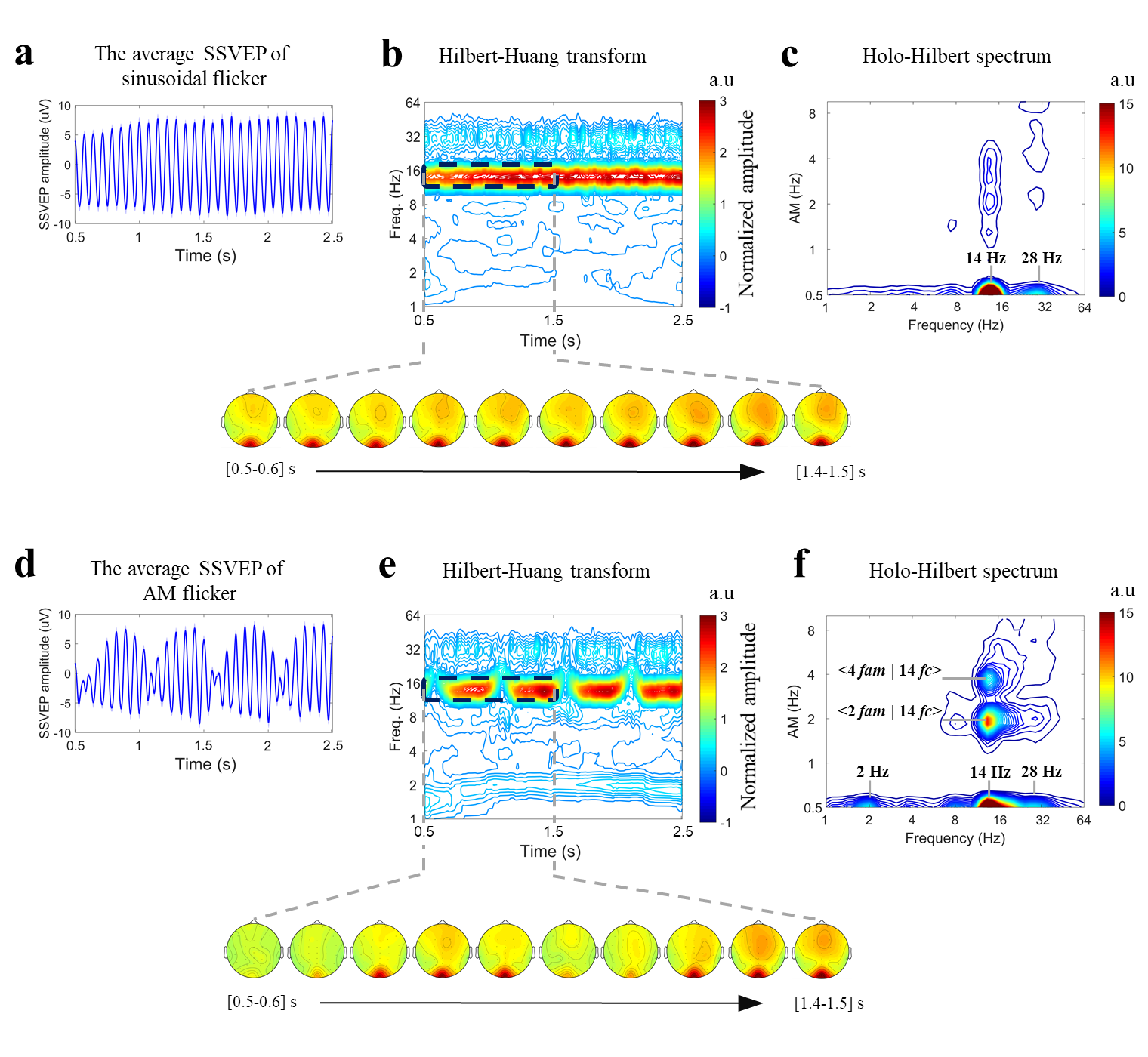 |
| --- |
| **Supplementary Figure S4.** (Experiment 1) The SSVEP elicited by sinusoidal and AM flicker in binocular stimulation, averaged across subjects for the Oz channel. **(a)** The average SSVEP response of sinusoidal flicker across subjects. **(b)** The Hilbert-Huang transform of SSVEP induced by sinusoidal flicker shows the strong amplitude response around the stimulus frequency (14 Hz). Ten topographies indicated the stable activities in occipital channels over time. Each topography corresponds to the collapsed amplitudes in a time window of 100ms. **(c)** Holo-Hilbert spectrum of SSVEP response induced by sinusoidal flicker. In two-dimensional frequency representation, Holo-Hilbert spectrum showed strong amplitude at the fundamental frequency (i.e., 14 Hz carrier) and its frequency-doubling (i.e., 28 Hz carrier). Some weak amplitude of 2 to 4 Hz modulating 14 Hz were observed. **(d)** The grand average SSVEP responses induced by AM flicker across subjects. **(e)** The Hilbert-Huang transform during AM flicker showed that the SSVEP amplitude was concentrated on 14 Hz and temporally modulated by 2 Hz (i.e. the frequency of envelope). Ten topographies indicated that occipital activity was clearly modulated by the phase of envelope (2 Hz), that is: 14 Hz induced stronger occipital activity in the peak of envelope and lower activity in the trough of envelope. In subfigure **b** and **e**, the x-axis is the number of data points; the y-axis represents in a dyadic frequency scale. **(f)** Holo-Hilbert spectrum of SSVEP response induced by AM flicker. In this spectrum, the SSVEP amplitudes at <2 *f_am_* \| 14 *f_c_*>, <4 *f_am_* \| 14 *f_c_*> were observed in a two-dimensional frequency spectrum. In addition, the amplitudes of carrier frequencies at 2 Hz, 14 Hz and 28 Hz were also observed in HHS at the 0.5 Hz y-axis. |

| 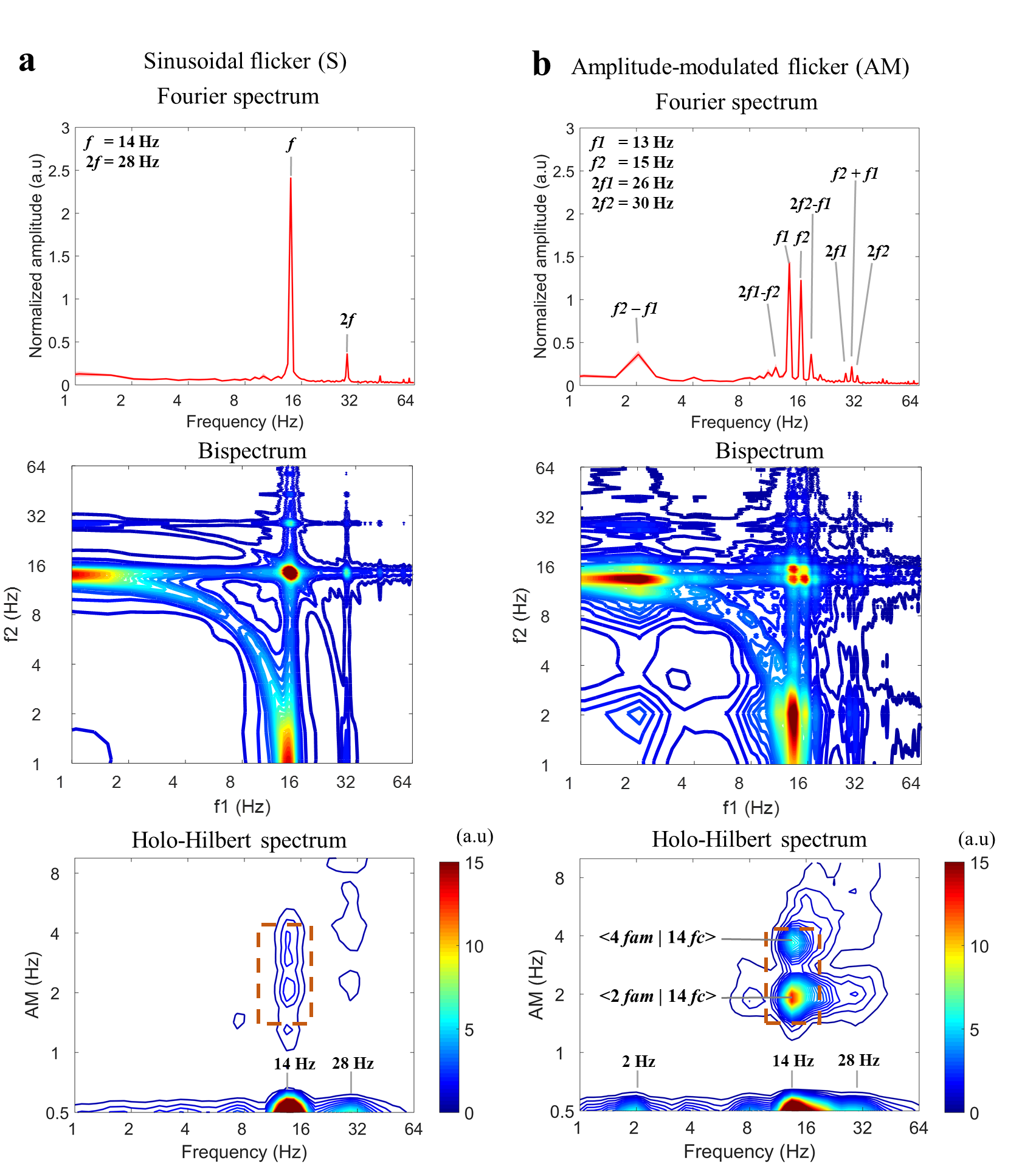 |
| --- |
| **Supplementary Figure S5.** The SSVEP amplitudes induced by AM and sinusoidal flicker at the Oz channel for binocular stimulation, averaged across participants. The SSVEP amplitudes observed in FFT (top panel), Bispectrum (middle panel), and Holo-Hilbert spectrum (bottom panel) during the sinusoidal flicker condition **(a)** and the AM flicker condition **(b)**. |

| 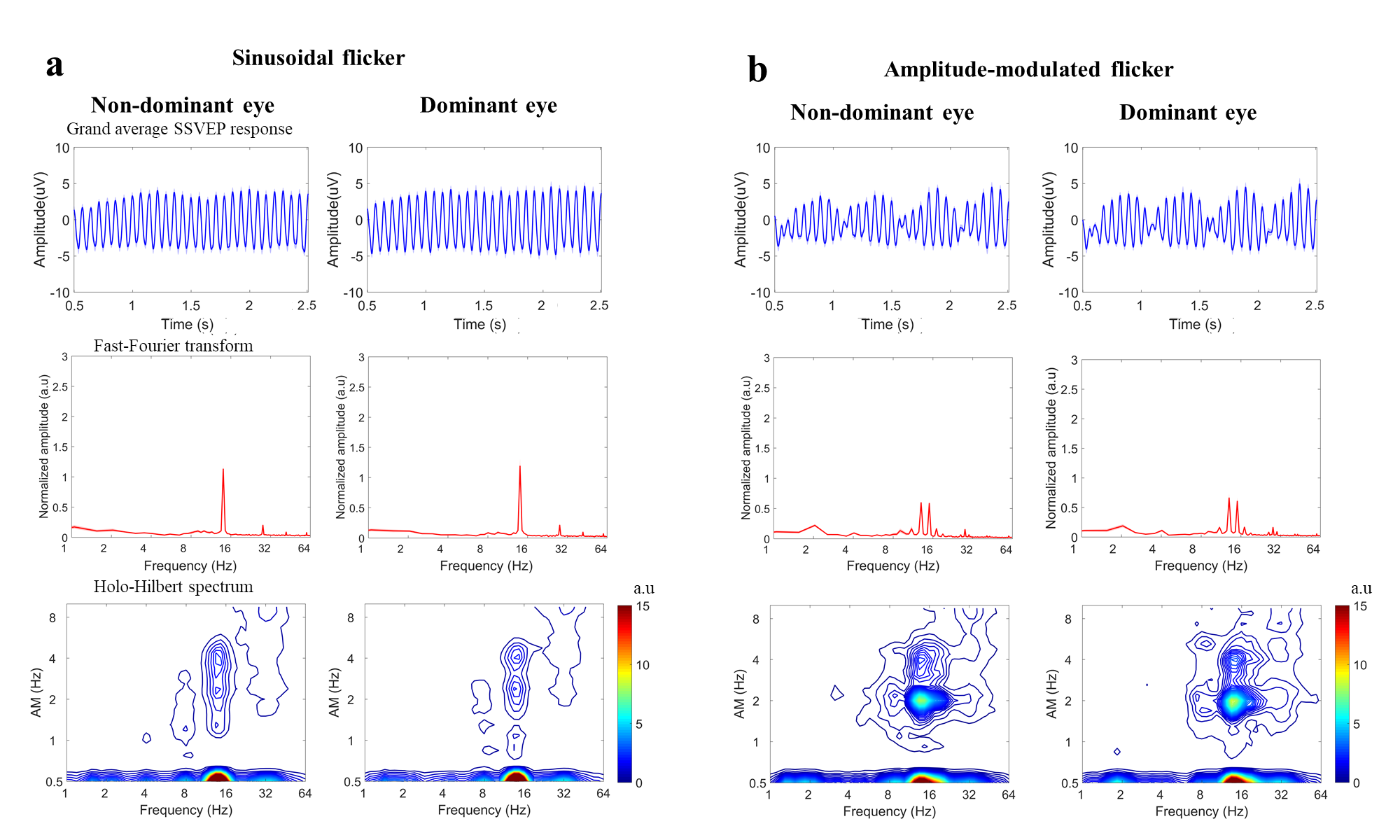 |
| --- |
| **Supplementary Figure S6.** (Experiment 1) The SSVEP responses of sinusoidal flicker and AM flicker in monocular stimulation, averaged across subjects at the Oz channel. **(a)** From top to bottom panels, SSVEP responses elicited by sinusoidal flicker in the non-dominant and dominant eye are shown in the time domain (top panels), frequency domain (FFT) (middle panels), and two-dimensional frequency spectrum in HHS (bottom panels). In FFT (middle panels), SSVEP amplitude showed the peaks at the fundamental frequency (i.e., 14 Hz*)* and a nonlinear component at frequency-doubling (i.e., 28 Hz). The x-axis represents the frequency and the y-axis is with an arbitrary unit of normalized amplitude. In two-dimensional frequency spectra (bottom panels), HHS showed strong amplitudes at the fundamental frequency (i.e., 14 Hz carrier) and its frequency-doubling (i.e., 28 Hz carrier). Some weak amplitude of 2 to 4 Hz modulating 14 Hz were also observed. The x-axis represents the carrier frequencies (*f_c_*) and the y-axis represents the amplitude modulation frequencies (*f_am_*). At 0.5 Hz y-axis, this x-axis is the summed amplitudes of carrier frequencies over time. The frequency axes represent dyadic frequency. **(b)** From top to bottom panels, SSVEP responses elicited by AM flicker in the non-dominant and dominant eye are shown in the time domain (top panels), frequency domain (FFT) (middle panels), and two-dimensional frequency spectrum (HHS) (bottom panels). In FFT (middle panels), the SSVEP spectrum showed a series of nonlinear intermodulation components, in which frequencies could be expressed by the equation: m**f_1_*±n**f_2_*, where *f_1_* (13Hz) and *f_2_* (15Hz) were the fundamental frequencies, and m and n represent positive integers. In HHS (bottom panels), the SSVEP spectra at <2 *f_am_* \| 14 *f_c_*>, <4 *f_am_* \| 14 *f_c_*> were observed in two-dimensional frequency spectrum. In addition, the amplitudes of carrier frequencies at 2 Hz, 14 Hz and 28 Hz were also observed in HHS at the 0.5 Hz y-axis. |

| 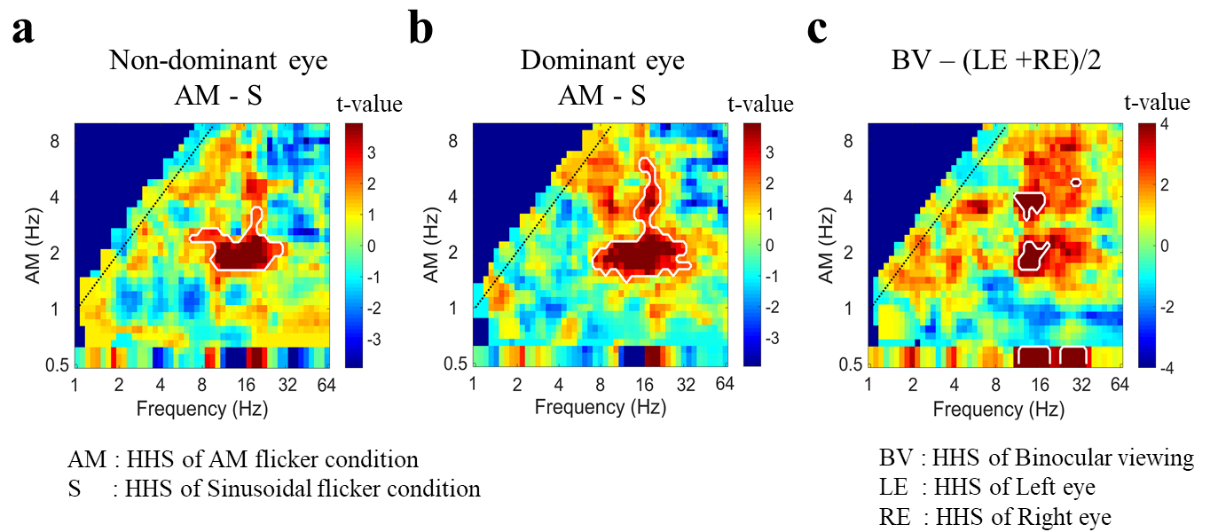 |
| --- |
| **Supplementary Figure S7.** (Experiment 1) The HHS contrast between SSVEP response elicited by AM and sinusoidal flicker at the Oz channel. **(a, b)** The HHS contrast between AM and sinusoidal flicker conditions in the non-dominant eye (**a**) and the dominant eye (**b**). The 2 Hz *f_am_* modulating wide carrier band around the fundamental frequency (14 Hz) was significantly different in both conditions (p<0.05, df=12, two-tailed, CBnPP test)**. (c)** HHS contrast of SSVEP responses between binocular viewing and averaging monocular viewing, in which HHS of SSVEP responses of the left eye and right eye were first summed then divided by 2 (i.e., (LE + RE)/2). The red area within the white contour shows the stronger amplitudes at <2 *f_am_* \| 14 *f_c_*> and its <4 *f_am_* \| 14 *f_c_*> in binocular stimulation compared to the averaged monocular eye (p<0.05, df = 12, two-tailed, CBnPP test). |

| 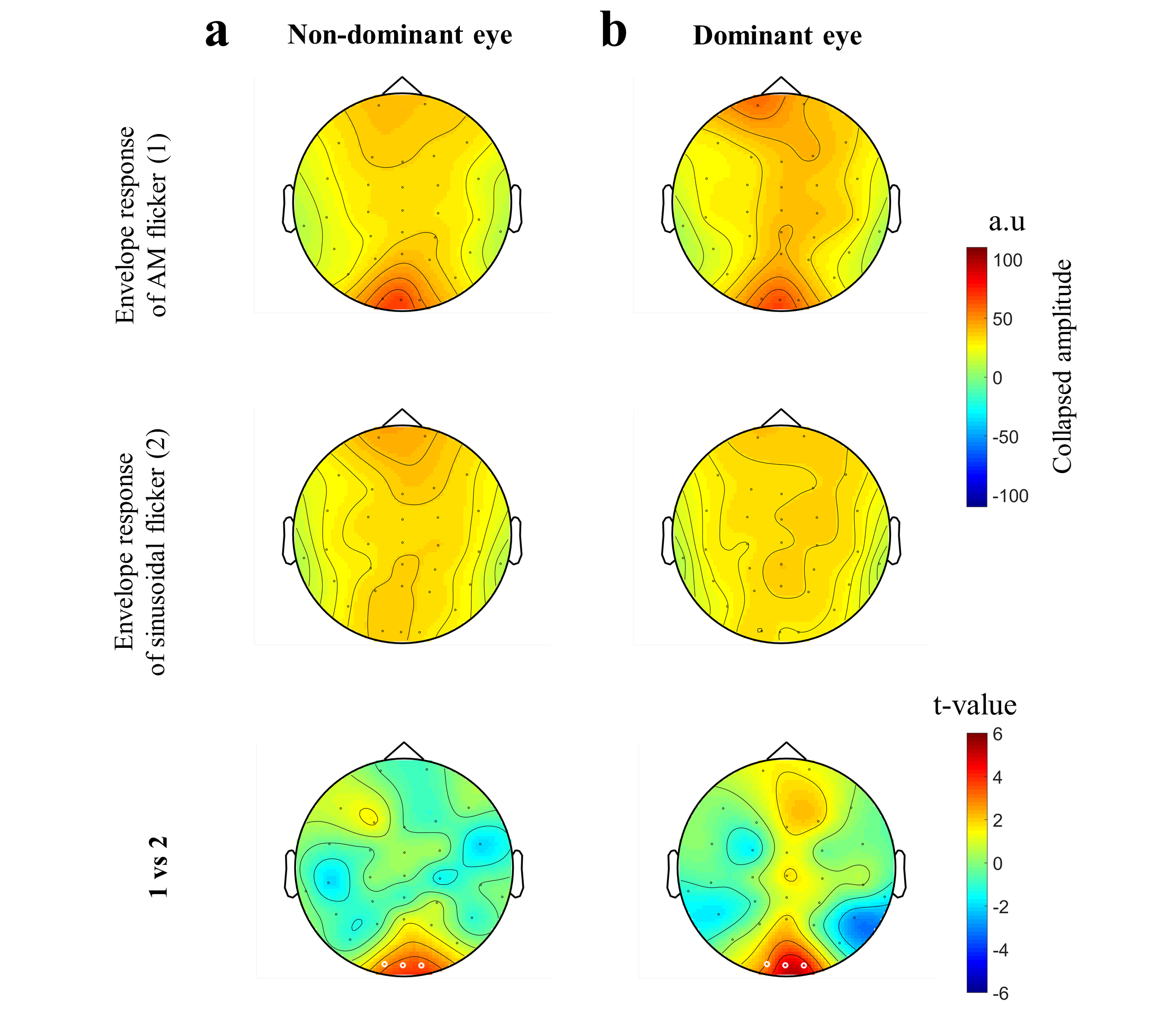 |
| --- |
| **Supplementary Figure S8.** (Experiment 1) SSVEP distribution of the envelope responses to monocular stimulation. **(a)** **The HHS topographies of envelope responses** in the non-dominant eye**.** In the AM flicker condition (top panel), **the HHS t**opography of the envelope during AM flicker denotes the collapsed amplitude with *f_am_* between 1.5 and 4.5 Hz, and f*c* from 12 to 16 Hz and it shows the strong amplitude distribution in the occipital channels. In the sinusoidal flicker condition (middle panel), the HHS topography did not show clear distribution of envelope-induced responses, since sinusoidal flicker did not induce much envelope response. Bottom panel, the topographic contrast between AM and sinusoidal flicker condition shows significant effects indicated by the concentric white circles in three occipital channels (p < 0.05 in the cluster-based non-parametric permutation test). **(b) Same as (a), but for the dominant eye.** |

| *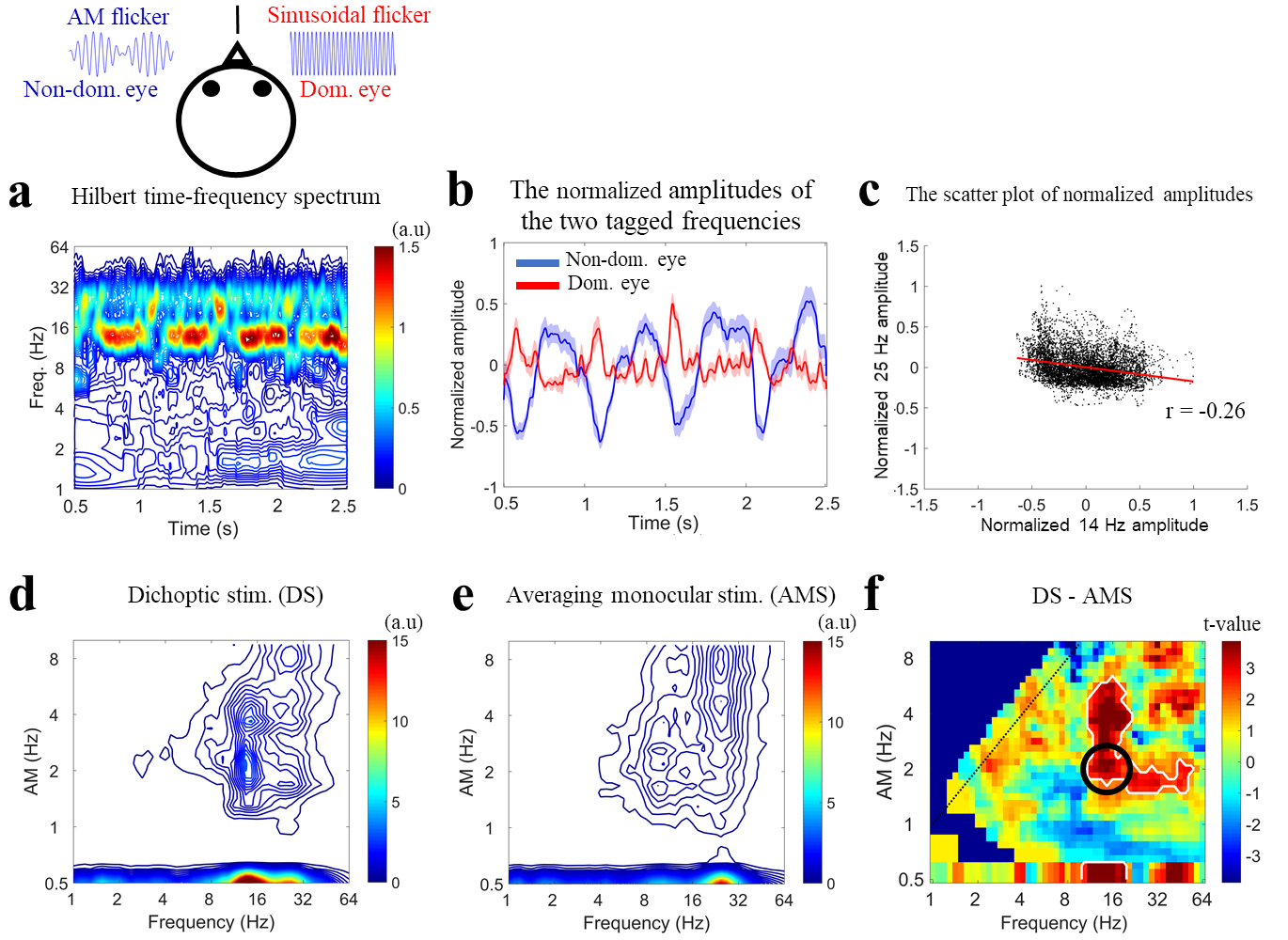* |
| --- |
| **Supplementary Figure S9.** (Experiment 2) The interocular correlation of SSVEP responses across subjects during dichoptic stimulation, in which 2 Hz modulating 14 Hz AM flicker is presented to the non-dominant eye and 25 Hz sinusoidal flicker is presented to the dominant eye simultaneously. **(a)** The spectral amplitude of SSVEPs during dichoptic stimulation were observed in Hilbert-Huang transform spectrum, averaged across subjects for the Oz channel. **(b)** The time course of the amplitude of two eyes’ tagged frequencies from the HHT spectrum. The red curve shows the time course of amplitude corresponding to the sinusoidal flicker (amplitude at 25 Hz), and the blue curve shows the time course of amplitude corresponding to the AM flicker (amplitude at 14 Hz). Red and blue shaded areas are standard errors of the mean. **(c)** The scatter plot of the amplitudes of the two tagged frequencies shows the strong negative correlation between the two eyes. Each dot corresponds to a 4 ms time bin in the time course. Data were pooled together across subjects. **(d)** The HHS of SSVEP response during dichoptic stimulation. **(e)** The HHS was calculated by averaging the HHS from each monocular stimulation (including left and right eye). **(f)** The contrast between the HHS from binocular viewing and the mean HHS from each monocular stimulation, showing the significant t-value at <2 *f_am_* \| 14 *f_c_*> (within the dark circle), <4 *f_am_* \| 14 *f_c_*> and <2 *f_am_* \| 25 *f_c_*> (white contoured area centered at the red area, p <0.05, df = 13, two-tailed, CBnPP test). |

**References**

1. Huang, N. E. *et al.* On Holo-Hilbert spectral analysis: a full informational spectral representation for nonlinear and non-stationary data. *Philos. Trans. R. Soc. A Math. Phys. Eng. Sci.* **374**, 20150206 (2016).

2. Tsai, F. F., Fan, S. Z., Lin, Y. S., Huang, N. E. & Yeh, J. R. Investigating power density and the degree of nonlinearity in intrinsic components of anesthesia EEG by the Hilbert-Huang transform: An example using ketamine and alfentanil. *PLoS One* **11**, 1–16 (2016).

3. Deering, R. & Kaiser, J. F. The use of a masking signal to improve empirical mode decomposition. in *Proceedings. (ICASSP ’05). IEEE International Conference on Acoustics, Speech, and Signal Processing, 2005.* **4**, iv/485-iv/488 Vol. 4 (2005).

4. Huang, N. E. *et al.* On instantaneous frequency. *Adv. Adapt. Data Anal.* **01**, 177–229 (2009).

5. Huang, N. E. *et al.* The uniqueness of the instantaneous frequency based on instrinsic mode function. *Adv. Adapt. Data Anal.* **05**, 1350011 (2013).

6. Swami, A., Mendel, C., Nikias, C. Higher-order spectral analysis (hosa) toolbox. Version 2, 3. (2000).

7. Iatsenko, D., McClintock, P. V. E. & Stefanovska, A. Nonlinear mode decomposition: A noise-robust, adaptive decomposition method. *Phys. Rev. E.* **92**, 032916 (2015).

8. Apostolidis, G. K. & Hadjileontiadis, L. J. Swarm decomposition: A novel signal analysis using swarm intelligence. *Signal Processing* **132**, 40–50 (2017).
